# Creation of distinctive Bax-lipid complexes at mitochondrial membrane surfaces drives pore formation to initiate apoptosis

**DOI:** 10.1126/sciadv.adg7940

**Published:** 2023-06-02

**Authors:** Luke A. Clifton, Hanna P. Wacklin-Knecht, Jörgen Ådén, Ameeq Ul Mushtaq, Tobias Sparrman, Gerhard Gröbner

**Affiliations:** ^1^ ISIS Pulsed Neutron and Muon Source, Science and Technology Facilities Council, Rutherford Appleton Laboratory, Harwell Science and Innovation Campus, Didcot, Oxfordshire OX11 OQX, UK.; ^2^ European Spallation Source ERIC, ESS, P.O. Box 176, SE-22100 Lund, Sweden.; ^3^ Department of Chemistry, Division of Physical Chemistry, Lund University, P.O. Box 124, SE-22100 Lund, Sweden.; ^4^ Department of Chemistry, University of Umeå, SE-90187 Umeå, Sweden.

## Abstract

Apotosis is an essential process tightly regulated by the Bcl-2 protein family where proapoptotic Bax triggers cell death by perforating the mitochondrial outer membrane. Although intensively studied, the molecular mechanism by which these proteins create apoptotic pores remains elusive. Here, we show that Bax creates pores by extracting lipids from outer mitochondrial membrane mimics by formation of Bax/lipid clusters that are deposited on the membrane surface. Time-resolved neutron reflectometry and Fourier transform infrared spectroscopy revealed two kinetically distinct phases in the pore formation process, both of which were critically dependent on cardiolipin levels. The initially fast adsorption of Bax on the mitochondrial membrane surface is followed by a slower formation of pores and Bax-lipid clusters on the membrane surface. Our findings provide a robust molecular understanding of mitochondrial membrane perforation by cell-killing Bax protein and illuminate the initial phases of programmed cellular death.

## Teaser

Bax initiates apoptosis by perforating mitochondrial membranes via formation of pores and extramembranous Bax-lipid complexes.

## INTRODUCTION

Programmed mammalian cell death, also denoted apoptosis, is essential for embryonic development, tissue homeostasis, and removal of harmful cells (*1*, *2*). Failure in its regulation can cause various pathological disorders including cancer (*3*, *4*). Upon activation of the intrinsic apoptotic pathway by intracellular stress, mitochondria become intimately involved in its further progression toward cellular death (*5*). During this process, the mitochondrial shell, its mitochondrial outer membrane (MOM), undergoes permeabilization, thereby enabling release of apoptotic factors such as cytochrome c that triggers an irreversible signaling cascade (*6*). To protect healthy cells from undesired clearance, this pathway is tightly regulated by the B cell lymphoma 2 (Bcl-2) protein family (*5*, *7*–*9*). Pro- and antiapoptotic members of this family meet at the MOM, and they arbitrate the cell’s fate there: intact membrane and survival versus permeabilization and death (*1*, *2*). Failure in this regulation process can cause various pathological disorders ranging from embryonal defects to cancer (*3*, *4*).

The most prominent multidomain members of the Bcl-2 family are the apoptotic Bcl-2–associated X protein (Bax) and the antiapoptotic Bcl-2 protein itself. Both proteins have the typical features of “tail anchored membrane proteins” with an amphitropic main globular fold and a single transmembrane (TM) domain for association to and penetration into the MOM target membrane (*5*, *6*). While Bcl-2 is insoluble and immersed in the membrane environment (*7*), Bax with a similar homology is soluble by hiding its TM domain inside its hydrophobic groove region (*8*). Bax is mainly found in the cytosol, but, upon activation by apoptotic stimuli, it exposes its TM domain, causing it to be massively recruited to the MOM, where it undergoes conformational changes, and, upon partial penetration into the membrane, lastly gives rise to membrane pores and lethal release of apoptotic factors (*9*–*12*). In healthy cells, pore formation by is prevented by retrolocation of transiently membrane-associated Bax back to the cytosol (*13*). In this recruitment process of Bax to the MOM, the mitochondria-specific lipid cardiolipin (CL) plays a driving role (*10*, *14*–*19*). While CL is very abundant at the inner mitochondrial membrane, the MOM has ca. only 4 mole percent (mol %) of its lipids as CL in most areas increasing to 20 mol % near the contact sites connecting MOM with the inner membrane (*20*, *21*). These regions with increased CL could allow localized amplification of apoptotic signals by activated Bax (*16*, *17*, *22*). However, high-resolution structures of membrane-bound Bax and time-dependent studies of the pore formation process are still lacking, despite recent progress on pore characterization by computational approaches (*22*) and by high-resolution electron paramagnetic resonance (EPR), microscopy, and fluorescence-based methods (*11*, *17*, *23*–*25*). In particular, in the absence of information on the organization of lipids in the Bax-induced pores and Bax clusters, the precise mechanism driving Bax membrane association and pore assembly remains quite unclear at the molecular level.

To obtain a molecular understanding of the ability of the Bax protein to permeabilize MOMs by recognizing them and creating pores, we studied the spatial and temporal organization of intact human Bax protein and membrane lipids during the pore formation process in MOM-like supported lipid bilayers (SLBs) containing varying amounts of CL, to unravel the key roles of CL in membrane association/activation of Bax, membrane perforation and subsequent pore formation, and cytochrome c release during apoptosis (*14*, *16*, *17*, *22*). To follow the molecular organization of Bax-induced pores and time evolution of membrane/Bax structures including individual components, we applied two complementary approaches: neutron reflectometry (NR) and attenuated total reflection Fourier transform infrared (ATR-FTIR) spectroscopy on SLBs (*26*, *27*). During recent years, NR with deuterium isotopic labeling has become a powerful technique for providing the structural envelope and compositional depth profile across a membrane to resolve the relative distribution of lipids and proteins (*28*–*30*), thus offering a unique way to determine the constituents of Bax-induced pores. To mimic the nonhomogenous CL abundance in vivo, MOM-like bilayers with varying CL contents (5 to 15 mol %) were used. In situ titration of Bax to supported bilayers enabled us to monitor the Bax-membrane interaction and reorganization of the structures as a function of time by determining the constituents of Bax-induced pores in the vertical dimension across the bilayer. In particular, changes in location and distribution of molecular components can then visualized as scattering length density (SLD) (*7*) and corresponding component volume fraction profiles (*31*). As a complementary method, ATR-FTIR provided valuable information on the kinetic and structural behavior of Bax and lipid matrix organization via exploiting protein-specific amide I infrared (IR) bands and the IR lipid acyl chain (CH) stretches (*27*–*30*).

We observed that, upon a rapid initial adsorption, Bax penetrates into the membrane on a slower time scale, causing perforation and deposition of Bax/lipid assemblies on the surface of the bilayer. The process occurs on a time scale of 2 to 5 hours, which is of the same order of magnitude as in vivo apoptosis (*32*). We further found that the initial kinetics of pore formation are dependent of the CL content of the bilayer. This type of spatial, temporal, and compositional information within a bilayer membrane is not accessible by other high-resolution approaches such as stimulated emission depleted (STED)/atomic force microscopy (AFM) (*17*), EPR/nuclear magnetic resonance spectroscopy (*15*, *19*, *33*, *34*), cryo–transmission electron microscopy (*35*, *36*), or x-ray crystallography (*37*), which probe other important structural aspects. The detailed view of Bax pore assembly with the discovery of Bax-lipid clusters obtained by our time-resolved NR approach provides a solid foundation to understand the membrane-destroying function of apoptotic multidomain members of the Bcl-2 family in general and opens up the way for overcoming their molecular inhibition in many tumors in the search for cancer therapies.

## RESULTS AND DISCUSSION

### Reorganization of the MOM in the presence of Bax

**Fig. 1.**
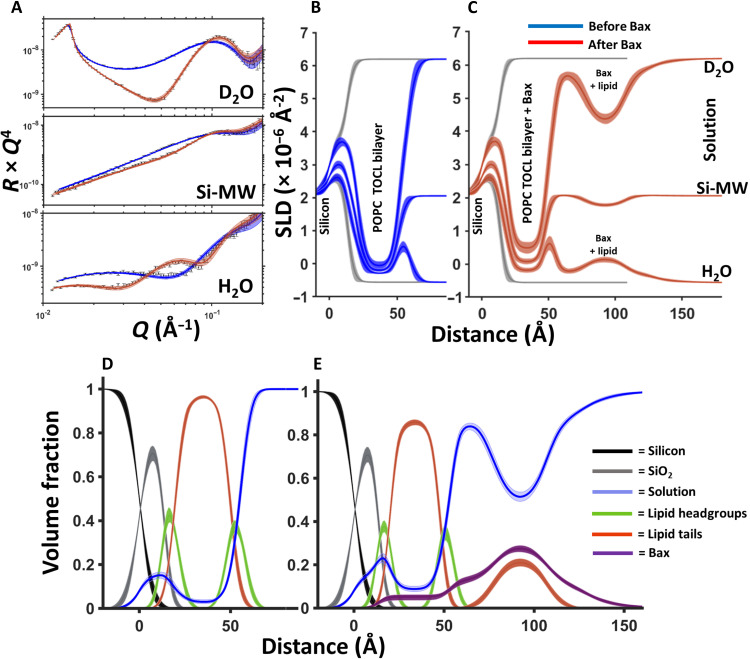
NR data showing Bax-induced redistribution of lipids from the membrane core to a surface protein-lipid complex during its apoptotic mechanism. NR data (error bars) and model data fits (lines) from a 90 mol % POPC–10 mol % TOCL SLB before (blue) and after (red) the interaction of natural abundance hydrogen h-Bax are shown in three differing solution isotopic contrast conditions being D_2_O, Si-MW, and H_2_O (**A**) buffer solutions. The SLD profiles and the model fits are shown for the surface structure before (**B**) and after (**C**) the h-Bax interaction. The corresponding component volume fraction profiles are shown before (**D**) and after (**E**) the h-Bax interaction as determined from the NR fits. Individual components are color-coded as indicated, with the Bax protein distribution in purple. Note that, after the interaction of the protein, there is a lower lipid content in the SLB and a distribution of lipid on the membrane surface. Line widths in the NR data fits represent the 65% confidence interval of the range of acceptable fits determined from Monte Carlo Markov chain (MCMC) error analysis, and the line widths in the SLD and volume fraction profiles represent the ambiguity in the resolved interfacial structure determined from these.

NR enabled us to profile the vertical distribution of molecular constituents in MOM-like bilayers in the presence of intact human Bax protein as seen in Fig. 1. Vesicle fusion was used to deposit a series of SLBs composed of a mixture of the zwitterionic phospholipid 1-palmitoyl-2-oleoyl-phosphocholine (POPC) and the anionic phospholipid tetra-oleoyl-CL (TOCL), to probe the role of CL present in the MOM (*20*), in Bax pore formation. Those type of lipid bilayers show the same basic behavior as intact MOMs (*10*, *18*) and are therefore used as established and reliable model biomembranes by many groups (*9*, *10*, *16*–*19*). Three individual bilayer systems were used with increasing CL content (5, 10, and 15 mol %) to represent the variation in CL abundance in different regions of the MOM varying from 4 up to 20 mol % at mitochondrial membrane contact sites (*20*).

Using 10 mol % CL containing bilayers as an example, Fig. 1 shows the corresponding NR data, model data fits, and the SLD profiles before and upon the interaction of Bax under steady-state conditions (several hours) at 37°C. NR results describe the distribution of the protein, lipid, and solution components across MOM models before and after Bax-mediated poration. Structural results are given as volume fraction versus distance profiles (see Fig. 1, D and E), that is, the relative amount of each component at a given region across the surface structure. The changes in these component distributions before and after the interaction of Bax with the MOM model yielded molecular level details on this protein’s membrane-perturbing function across the membrane.

Before the interaction of Bax the surface coverage of the MOM-like bilayers was 98 ± 1 volume percent (volume %) lipid (see Table 1), after the interaction of the protein the lipid content of the SLB had been reduced to 86 ± 2 volume % with an increase in the water content of the lipid tail region of the SLB from 2 ± 1 volume % to 9 ± 1 volume %, with 5 ± 2 volume % protein also appearing in this region (Fig. 1, C and E). In addition, the SLB was also seen to become thinner with the SLB tail thickness reducing from ~30 to ~28 Å (see Table 1). Together with the observed increase in water and protein within the SLB, these data are suggestive of the formation of pores within the membrane, an activity that is directly related to Bax’s role as an initiator of the biochemical stages of apoptosis (*17*, *23*, *38*). The activation of Bax is here driven by direct contact with CL containing membranes, in agreement with recent studies on direct lipid dependent activation of Bax (*15*, *16*, *19*, *39*). Any inactivated Bax remains in solution and is not detected in the NR experiment that only probes the surface region.

**Table 1.** The resolved structural components before and after the interaction of Bax with SLB composed of POPC–10 mol % TOCL.*

	Average lipid area per molecule (Å^2^)	Tail thickness (Å)	Tail composition (volume %)	Headgroup thickness (Å)	Outer headgroup composition (volume %)	Membrane surface Bax/lipid complex thickness (Å)	Bax surface layer composition (volume %)
**POPC: CL (90:10 mol %)**	64.5 Å^2^ (63.8 Å^2^, 65.2 Å^2^)	29.8 Å (29.4 Å, 30.1 Å)	Lipid, 98% (97%, 99%)	5.7 Å (5.4 Å,6.4 Å)	Headgroup, 83% (81%, 89%)	–	–
			Solution, 2% (1%, 3%)		Water, 17% (14%, 21%)		
**POPC: CL (90:10 mol %) + h-Bax**	69.7 Å^2^ (68.7 Å^2^, 70.2 Å^2^)	27.6 Å (26.9 Å, 29.0 Å)	Lipid, 86% (85%, 88%)	6.5 Å (6.2 Å, 6.6 Å)	Lipid, 65% (63%, 68%)	**1**, 23.4 Å (22.2 Å, 24.9 Å)	Protein lipid distribution composition:
			Protein, 5% (3%, 6%)		Protein, 5% (3%, 6%)	**2**, 29.7 Å (27.1 Å, 31.2 Å)	1, Protein, 12% (10%, 14%); water, 88% (86%, 90%)
			Solution, 9% (8%, 10%)		Solution, 30% (27%, 34%)	**3**, 23.4 Å (22.2 Å, 24.9 Å)	2, Protein, 30% (27%, 32%); lipid, 17% (16%, 18%); solution, 53% (51%, 55%)
						Total: 76.4 Å (75.2 Å, 77.7 Å)	3, Protein, 12% (10%, 14%); water, 88% (86%, 90%)

*Values in parentheses represent the 65% confidence intervals determined from MCMC resampling of the experimental data fits.

The interaction with Bax produced a symmetrical protein-lipid complex on the membrane surface. The structure was composed of a complex layer of protein and lipid between two layers of protein. The amount of lipid removed from the MOM models due to poration and membrane thinning was equal to that found in the Bax/lipid clusters formed on the membrane surfaces (see Table 1), strongly supporting a mechanism of membrane restructuring during the Bax-induced poration process. Together, these results show that Bax is able to shuttle lipids from the membrane interior to the membrane surface during its pore-forming activity. Complementary ATR-FTIR measurements on the same MOM models showed broadening of the lipid CH_2_ stretches as a result of the interaction of Bax (figs. S5 and S6). This is suggestive of an increasingly disordered membrane in agreement with the partial lipid shuttling activity shown by NR.

Those changes in the interfacial membrane structures are representative for all samples with varying CL content. Measurements and corresponding analysis plots for SLBs containing 5 and 15 mol % CL are shown in the Supplementary Materials (fig. S1 and S2) and resolved the same membrane surface protein-lipid cluster as was seen for the 10 mol % CL datasets. In addition, there was a noticeable trend in the NR data where the amount of lipid removed from the SLB to form pores was proportional to the initial CL content (see Table 2). Nevertheless, Bax-induced membrane thinning was fairly consistent across all samples with a ~2- to 3-Å decrease in bilayer thickness observed at equilibrium Bax binding (Table 1 and tables S1 to S3).

**Table 2.** The relationship between SLB TOCL concentration and lipid loss during Bax driven membrane disruption.*

SLB TOCL content	5 mol %	10 mol %	15 mol %
**SLB tail coverage before Bax (volume %)**	99% (98%, 100%)	98% (97%, 99%)	99% (98%, 100%)
**SLB tail coverage after Bax (volume %)**	91% (90%, 92%)	86% (85%, 88%)	81% (80%, 82%)
**Lipid removed through pore formation (volume %)**	8% (6%, 10%)	12% (9%, 14%)	18% (16%, 20%)
**Bax in central protein/lipid clusters**	29% (27% 32%)	30% (27%, 32%)	35% (32%, 37%)

*Tables of NR resolved structural parameters from the interaction of Bax with SLBs containing 5 and 15% are given in tables S1 and S2, respectively. Values in parentheses represent the 65% confidence intervals determined from MCMC resampling of the experimental data fits.

### Schematic model of membrane perforation by Bax

As the mechanism of redistribution of the lipid from the membrane to the membrane surface by Bax provides insight into the role of membrane lipids in the initial stages of apoptosis, NR measurements on the interaction of Bax with a POPC–10 mol % TOCL SLB were repeated using deuterium-labeled protein. The advantage of using deuterated d-Bax is that the d-protein will be contrast matched (i.e., invisible) in the D_2_O contrast condition in NR measurements (*7*), giving one dataset that only describes the structure of the SLB and the lipid components. These data (fig. S3) provided independent confirmation that the interaction of Bax with the model membrane caused a redistribution of lipids from the SLB to the membrane surface and again showed that the lipid component that moved to the membrane surface matched the amount lost from the SLB during pore formation and bilayer thinning. The corresponding H_2_O contrast (fig. S3), on the other hand, confirms unambiguously the presence of d-Bax in the lipid-protein clusters on the membrane surface.

**Fig. 2.**
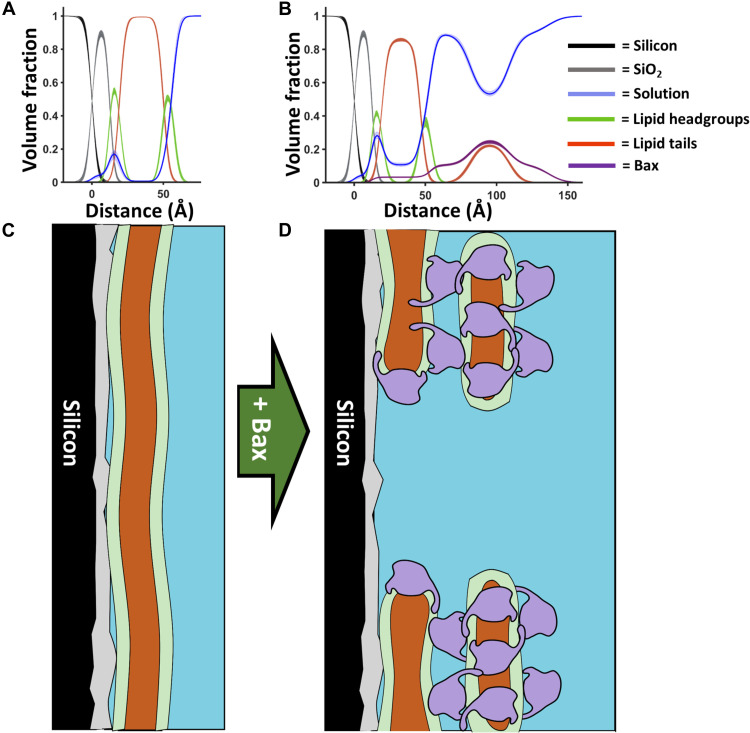
A model for Bax pore formation. Component volume fraction profiles from before (**A**) and after (**B**) the interaction of Bax with mitochondrial model membranes (in this case, d-Bax to a 90 mol % POPC–10 mol % TOCL SLB; see fig. S3). Below these are schematics (**C **and **D**) of how this component distribution in the *Z* direction may relate to the protein and lipid distributions across the surface before and after Bax interaction. The distribution of Bax across the membrane (D) is shown as a representation of how many layers of this protein, depicted as the envelope of its structure dimensions (*8*), can be distributed across the porated membrane based on NR results. This model comes from consideration of the data presented here and in previous SLB studies using atomic force microscopy (*17*).

The thickness profile of the membrane surface protein-lipid complex was suggestive of a structure that was three times the width of Bax in its folded solution state (*40*). We have proposed a model of the SLB surface structure (Fig. 2) where Bax forms a pore within the SLB, around which there is a protein lipid complex composed of three distinct layers: one continuing mainly protein between the SLB and the second layer containing both proteins and lipid tails, and third layer containing mainly protein. Although the lipid polar headgroups are not resolved per se due to the low surface coverage (~15 volume %) of the surface complexes and low contrast to the protein, we interpret this structure as essentially small lipid bilayer patches in which Bax is found in both the hydrophobic core layer and the outer headgroup layers, as illustrated in Fig. 2D. Bax seems to perforate the MOM by sequestering membrane lipid components into Bax/lipid clusters deposited above the bilayer to make space for the pores. The formation of this membrane surface structure is consistent with the ring structures found to enclose Bax-induced pores in previous topological AFM studies (*17*). In that study, activated Bax formed protruding Bax clusters (like coronas) around 4 nm above the SLB, a scenario that is in well agreement with our Bax-lipid complexes and their thickness distribution as seen in Fig. 2. In our model, Bax forms pores at the membrane level with a low fraction of Bax residing inside the membrane compared to that found on the membrane surface (Fig. 2B and tables S1 to S3), which is partially in agreement with previous lipidic pore models (*34*, *38*). Our model clearly supports and fundamentally expands recent superresolution microscopy and cryo–electron microscopy studies showing in human cells the formation of green fluorescent protein–labeled Bax clusters with higher molecular organization near mitochondria (*25*). Even for Bak, a close relative of Bax, disordered clusters of Bak dimers were found to generate lipidic pores (*41*). However, in contrast to these and previous studies, our results show unambiguously that the lipids excluded to create pores form a part of the proteolipid clusters observed above the SLB.

### Real-time kinetics of Bax-induced permeabilization of MOMs

In general, to create pores, Bax has to undergo structural changes upon membrane contact to form dimers, which then oligomerize into protein clusters to perforate the membrane (*23*, *25*, *40*, *41*). To unravel in depth the molecular principles and determinants by which Bax executes this complex process, particularly recognition and reorganization of the MOM, for enabling further apoptotic steps, we carried out a series of experiments to study the kinetics and dynamics of Bax interactions and their dependence on the amount of CL in the target membranes. Therefore, a combination of NR and ATR-FTIR was used to give time-dependent structural and mechanistic insight into the kinetics of the Bax/SLB interaction.

**Fig. 3.**
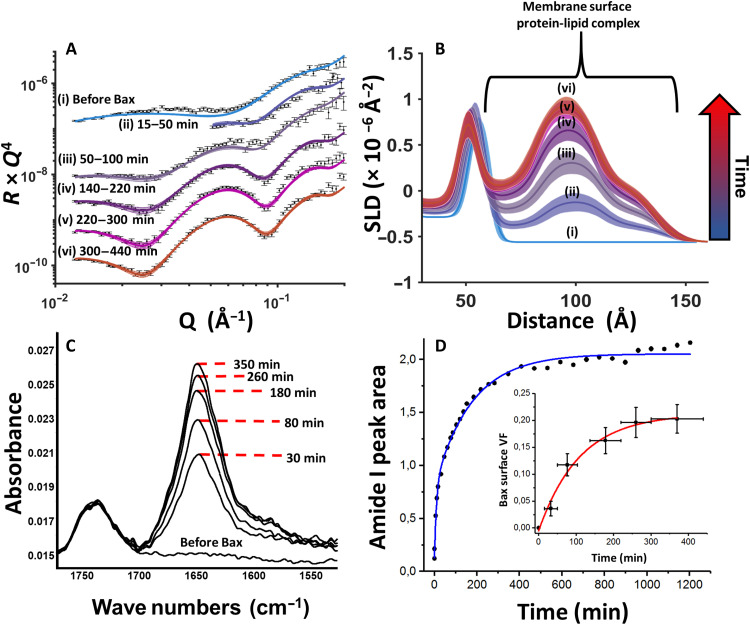
Time-dependent structural data based on the interaction of Bax with model MOMs. NR data (error bars) and model data fits (lines) obtained in a H_2_O buffer from a POPC–10 mol % TOCL SLB before and at different times after introduction of deuterated d-Bax into the solid-liquid cell containing the bilayer (**A**). The SLD profiles obtained from model fitting of these data reveal the increasing coverage of Bax on the membrane surface with increasing time (**B**). Complementary ATR-FTIR measurements on the same experimental system show the increasing size of the amide I band indicating the accumulation of Bax at the membrane containing solid-liquid interface (**C**). Last, a comparison of the proteins surface volume fraction (VF) (crosses) and amide I peak areas (red) versus time obtained from NR and ATR-FTIR measurements, respectively, showing the strong agreement between in the two techniques on the kinetics of the Bax/membrane interaction, suggesting two time-constant kinetic processes during Bax-MOM poration (**D**). For NR, each point on this plot represents an individual dataset, and the error bars on the *x* axis represent the range of time over which the dataset was collected, while the *y* errors represent the ambiguity on protein volume fraction determined by MCMC resampling of the model-to-data fits. Collection times for each ATR-FTIR dataset were ~80 s.

Monitoring the interaction of deuterated d-Bax with a fully hydrogenous (h-)membrane in a H_2_O buffer solution allowed collection of time-dependent structural NR data that were highly sensitive to changes in the protein distribution across the surface. These data, shown in Fig. 3A, showed an increasing surface coverage of the full, three-layer, protein-lipid complex on the SLB surface against time (Fig. 3B). Independent measurements by ATR-FTIR using both d- and h-Bax (described in the Supplementary Materials) probed the increase in the protein amide I band of Bax against time (Fig. 3C), which gave an independent measurement of the accumulation of protein within the surface region containing the SLB model of the MOM (*7*, *42*, *43*). A comparison between the growth of the amide I adsorption band (Fig. 3C) and time-resolved NR measurements showed strong agreement (Fig. 3), suggesting that the majority of protein is located on the membrane surface only during the pore-forming process.

Combined kinetic analysis of the ATR-FTIR and time-resolved NR data showed that, first, there is an initial rapid adsorption and accumulation of Bax on the membrane surface, the rate of which decreases over time, similar to Langmuir isotherm adsorption behavior, and, second, a slower protein-lipid complex formation and perforation process. Fitting of the FTIR data with a two-exponential kinetic model (blue line in Fig. 3D) provided a 10 ± 2 min time constant for the fast process and ca. 175 ± 15 min for the slower process. Analysis the NR data (red line) data for the initial fast process could not be obtained because of the restrictions in the time resolution of the NR technique, but a time constant of 110 ± 30 min was obtained for the slower process, a value that is in quite good agreement with the FTIR results despite different sample environments and sample batches. Studies using fluorescence-labeled Bax overexpressed in intact cells observed a heterogeneous Bax cluster formation near mitochondria on a scale of 100 ± 60 min upon the initial Bax recruitment to mitochondria (*25*).

Together, these data suggest that the formation of the complex protein-lipid surface assemblies is commensurate with the Bax interaction with the SLB rather than a stepwise buildup of the three protein-containing layers on the membrane surface. Qualitatively, the two processes that we directly identified here by NR reflect earlier studies that suggest that, in the first phase, Bax becomes activated upon initial membrane contact and partially inserts to generate dimers under structural rearrangements. Those dimers are then a prerequisite for forming supramolecular pore structures of Bax at the MOM on a slower time scale to initiate further apoptotic activities (*17*, *34*, *44*). This later process occurs during several hours as monitored in our NR experiments in real-time that agrees well with times reported for in vivo cell death to happen (*32*): early apoptosis, a few hours; and late apoptosis, 6 to 24 hours.

### Role of CL in driving Bax-mediated membrane activities

The mitochondria-specific lipid CL plays a key role during apoptosis in facilitating recruitment of Bax to the MOM, the subsequent insertion of Bax into the membrane, and its final perforation (*10*, *14*, *16*, *19*). The distribution of CL across the MOM is quite inhomogeneous with around 4 mol % of CL in areas between contact sites and increases (>20 mol %) near contact sites (*20*, *45*). On the basis of the previous work on Bax-induced membrane leakage that occurs in a CL-dependent manner, we were interested in studying the time dependence of Bax/lipid assembly formation and outcome of membrane reorganization for MOM bilayers with varying CL content reflecting the different lipid environment at the MOM.

**Fig. 4.**
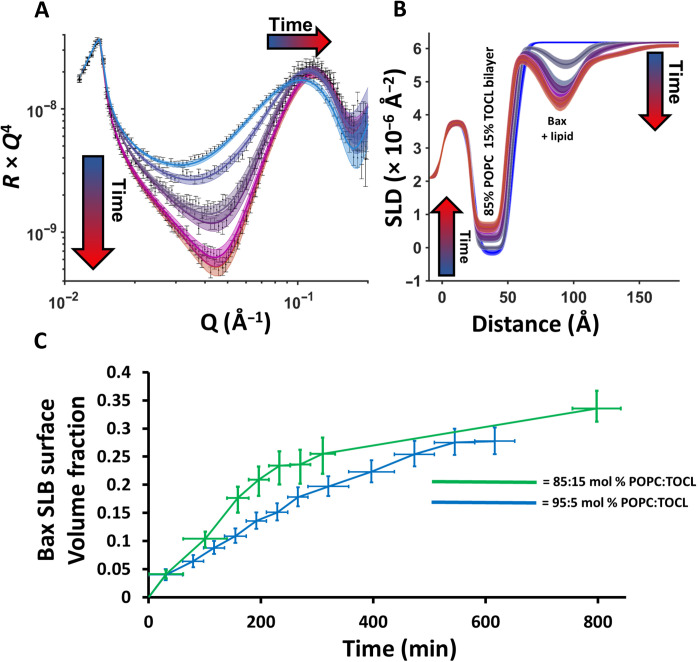
Time-resolved NR data. NR experiment at different time intervals revealed that pore formation and membrane surface protein-lipid complex formation increase proportionally with time and that the kinetics of the membrane reorganization process is positively correlated to bilayer CL concentration. Time-resolved NR data (error bars) and model data fits (lines) from a POPC–15 mol % TOCL SLB during the interaction of h-Bax in a D_2_O buffer solution are shown (**A**) as are the changes in the SLD profiles of the surface assemblage against time (**B**; depicted from blue to red) determined from model fitting. Note how the increasing SLD of the bilayer, depicting lipid loss, is commensurate with decreasing SLD, i.e., increasing coverage, of the Bax/lipid surface complex. Lines widths on the fits/SLD profiles depict the ambiguity in the resolved structure from MCMC error estimation. A comparison of the change in the fraction of Bax protein in the membrane surface complexes (in the middle layer) from NR data from a series of MOM models with differing CL content (**C**). Each data point on this plot represents an individual NR dataset, and the error bars on the *x* axis represent the range of time over which the dataset was collected, while the *y* errors represent the ambiguity on protein volume fraction determined by MCMC resampling of the model-to-data fits. Time-resolved NR data and fits for the interaction of Bax with the POPC–5 mol % TOCL bilayer are given in fig. S4.

Therefore, time-resolved NR data were used to examine the relationship between the pore and membrane surface protein-lipid complex formation and the concentration of CL in the MOM model. Figure 4 (A and B) shows time-resolved NR data obtained during the interaction of h-Bax with the 15 mol % TOCL–containing MOM model obtained in a D_2_O buffer solution. NR datasets obtained in D_2_O are sensitive to both the lipid and h-protein distributions across the surface (*26*). These data could be best fitted to models that were a scale ratio between the surface structure before the interaction of Bax and the structure at equilibrium Bax binding. This produced time-dependent structural changes where lipid removal from the SLB and membrane surface protein-lipid complex formation were proportional. This suggests that either individual pores in the SLB grow larger over time and/or that the number of pores increases. The direct correlation between pore and surface complex formation gives further evidence that the membrane surface protein-lipid complex is a by-product of pore formation.

The time dependence of the Bax interaction with the SLBs appeared to be strongly correlated to the concentration of CL in the membrane. Figure 4C shows a comparison of the change in membrane surface Bax volume fraction (from the highest density middle layer of the complex) from the interaction of the protein with the 15 and 5 mol % TOCL bilayers. These data suggests that the rate of interaction of Bax with the membrane is CL dependent. The same correlation with CL abundance was also observed for the absolute amount of lipids and Bax removed from the bilayer and deposited as protein/lipid complexes onto the membrane surface (see Table 2 and tables S1 to S3). The final coverage of Bax on the membrane surface was similar in all measurements with the exception of a minor increase in the Bax volume fraction in the central protein/lipid region of the surface clusters with higher MOM model CL concentrations and therefore lipid removal (see Table 2). On the basis of the results presented here, it is clear that the MOM CL content determines the rate of Bax recruitment to the membrane and the total amount of lipid removed by Bax into the membrane surface protein/lipid clusters.

Our results bridge a number of previous studies on the initial stages of apoptosis. Bleicken *et al.* (*9*) demonstrated that Bax caused budding from the membrane surface, while Salvador-Gallego *et al.* (*17*) showed the formation of defined membrane surface structures during the protein’s apoptotic activity with clusters surrounding pores. In addition, Bax assemblies into large ring-like structures were reported (*46*). The structural results shown here are in agreement with both findings, however, showing that the membrane surface material is composed of Bax/membrane lipid clusters that are formed as a direct result of membrane poration. Westphal *et al.* (*11*) proposed an “in-plane model” for the Bax interaction with the MOM where Bax lies flat on the membrane surface with only partial intercalation of the protein into the hydrophobic core of the MOM. Here, we observe a relatively small volume fraction of Bax within the MOM models after poration, with the vast majority of protein found on the membrane surface as a helix rich protein (fig. S8). We additionally observed a distribution of Bax and lipid on the membrane surface that is approximately three times the thickness of the protein structure dimensions (*8*). Because Bax has to oligomerize to create pores (*12*, *25*, *47*), our findings suggest a vertical Bax clustering on the membrane surface when extracting lipids from the MOM during its pore-forming activity. The low relative amount of Bax observed within the MOM models here also agrees with previous findings by Qian *et al.* (*48*), which suggested that Bax induces membrane-stabilized lipidic pores.

The presence of CL is a prerequisite for Bax to target the MOM and perforate it (*10*, *14*, *16*, *19*). From our data, we cannot distinguish any CL preference in the lipid removal into proteolipid complexes (see Figs. 3 and 4 and Table 2), but we can clearly observe that CL abundance determines the rate of Bax recruitment and the total amount of lipids deposited to the membrane surface. The amount of Bax found within the protein/lipid clusters on the membrane surface was less clearly correlated with the CL content (Table 2). However, there was, in the case of the 15% CL sample, some evidence that, with more lipid extracted from the membrane, the amount of Bax in the surface complexes increased. The general behavior that we have seen here is that Bax activity and its pore formation kinetics are strongly dependent on CL abundance, but the underlying molecular mechanism of Bax action remains the same. A behavior also reflected in leakage assays with varying CL contents (*10*, *15*, *16*, *18*, *19*).

From a biological perspective, it could be important that lipids get removed into clusters located physically away from the membrane pores to enable a quick release of cytochrome c via Bax-formed MOM pores into the cytosol to initiate further irreversible apoptotic steps (*14*, *22*). Our observed CL dependence of this process together with the inhomogeneous CL distribution across the MOM in vivo (*20*) presumably reflects two apoptotic mechanisms to regulate levels of membrane-associated Bax and its perforation activity without the involvement of Bcl-2 homology 3 (BH3) effectors from the Bcl-2 family, as recently proposed (*16*). CL could provide selective targeting and activation of Bax that would provide amplification of localized apoptotic signaling at CL-enriched MOM areas, a kind of “hotspot” near contact sites (*16*, *49*). In addition, during apoptosis, a major redistribution of CL from the inner CL-rich mitochondrial membrane [> 20 mol %; (*20*)] would further increase CL levels in the MOM (*50*). This could further accelerate Bax association and perforation activity to release cytochrome c quickly while increasing Bax levels to ensure a quick removal of CL into extramembranous Bax/lipid clusters. This would prevent any resequestering of cytochrome c into the CL complexes that exist at the inner mitochondrial membrane under normal conditions and ensure further release of cytochrome c into the cytosol to initiate further irreversible apoptotic steps (*14*, *22*). As shown by various groups, Bax induced pores in MOM-like bilayers as we used here, even in the absence of BH3 activators, sufficient in size to release cytochrome c (*16*, *19*, *22*).

Apoptosis initiated at the mitochondrial surface is a ubiquitous mammalian cellular process. The complex structural and mechanistic findings detailed in this study yield unique and direct structural evidence of the involvement of lipids in the structures formed during the membrane-perturbing mechanism of Bax, which initiates apoptosis in cells. These results give precision structural and kinetic details on Bax remodeling of the MOM and provide an explanation of the overlapping events of pore formation and lipid complexation. Thus, they reflect Bax activity in mitochondria during apoptosis, demonstrating how apoptotic Bcl-2 proteins such as Bax remodel the supramolecular architecture of the MOM shell to further drive apoptosis toward its ultimate goal, the removal of doomed cells.

## MATERIALS AND METHODS

### Material

#### Preparation of protonated (h-Bax) and deuterated (d-Bax) protein

The methods for expression and purification of Bax were accomplished by using previous procedures (*51*). Deuteration (>90%) of Bax was performed by using the following medium recipe: 1 liter of M9 medium containing 13 g of KH_2_PO_4_, 10 g of K_2_HPO_4_, 9 g of Na_2_HPO_4_, 2.4 g of K_2_SO_4_, 2 g of NH_4_Cl, 2.5 ml of MgCl_2_ (2.5 M stock), 1 ml of thiamine (stock, 30 mg/ml), 2 g of glucose (nondeuterated), and 2 g of NH_4_Cl (nondeuterated), supplemented with trace elements (1 ml of 50 mM FeCl_3_, 20 mM CaCl_2_, 10 mM MnCl_2_, 10 mM ZnSO_4_, 2 mM CoCl_2_, 2 mM CuCl_2_, 2 mM NiCl_2_, 2 mM Na_2_MoO_4_, and 2 mM H_3_BO_3_ per liter of medium), carbencillin (100 μg/ml), and chloramphenicol (34 μg/ml). The media were adjusted to pH 6.9 and sterile-filtered before use.

### Methods

#### NR measurements

NR measurements were performed on the white beam SURF (*52*) and OFFSPEC (*53*) reflectometers at the ISIS Neutron and Muon Source (Rutherford Appleton Laboratory, Oxfordshire, UK), which use neutron wavelengths from 0.5 to 7 Å and 1 to 12 Å, respectively. The reflected intensity was measured at glancing angles of 0.35°, 0.65°, and 1.5° for SURF and 0.7° and 2.0° for OFFSPEC. Reflectivity was measured as a function of the wave vector transfer, *Q_z_* [*Q_z_* = (4π sin θ)/λ, where λ is wavelength and θ is the incident angle]. Data were obtained at a nominal resolution (*dQ*/*Q*) of 4.0%. The total illuminated sample length was ~60 mm in all instruments. Measurement times for a single reflectometry dataset (~0.01 to 0.3 Å^−2^) were 40 to 60 min. Data collection times for kinetic datasets varied and can be seen as the *x* error bar on Fig. 3D (inset) and Fig. 4C.

Details of the solid-liquid flow cell and liquid chromatography setup used in the experiments described here are previously described (*54*). Briefly, solid-liquid flow cells containing piranha acid cleaned silicon substrates (15 mm by 50 mm by 80 mm with one 50 mm by 80 mm polished to 3-Å root mean square roughness) were placed onto the instrument sample position and connected to instrument controlled high-performance liquid chromatography pumps (Knauer Smartline 1000) that enabled programmable control of the change of solution isotopic contrast in the flow cell. The samples were aligned parallel to the incoming neutron beam with the beam width and height defined using two collimating slits before the sample position. The sample height was aligned in such a way that the neutron beam was centered on the middle of the sample surface.

#### Lipid membrane deposition

Initially, the clean silicon substrates were characterized by NR in D_2_O and H_2_O buffer solutions. Then, freshly sonicated lipid vesicle solutions (0.2 mg/ml) were introduced into the cells in the experiment buffer solution of 20 mM tris (pH 7.2), 150 mM NaCl, and 2 mM CaCl_2_, and the samples incubated at 37° ± 1°C for ~30 min before the non–surface-bound vesicles were removed by flushing the cells with 15 ml (~5 cell volumes) of the same buffer solution before a solution of pure D_2_O was flushed into the cell. The change in osmotic gradient across the lipid vesicles bound at the solid-liquid interface caused these to rupture, thereby forming high quality (i.e., high coverage) SLBs at the solid/liquid interface. The resulting SLBs were characterized by NR under three solution isotopic contrast conditions being D_2_O, silicon-matched water (Si-MW; 38% D_2_O), and H_2_O buffer solutions (CaCl_2_-free).

#### Bax interaction

Once characterization of the SLB was complete, the sample surface was placed in the correct solution isotopic contrast (D_2_O for h-proteins and H_2_O for d-proteins) and ~6 ml of a Bax solution (0.1 mg/ml) was injected into the flow cell (the cell volume is 3 ml) either by hand (SURF) or using a syringe pump (OFFSPEC, AL1000-220, World Precision Instruments, Hitchin, UK). In most cases, the interaction of the protein with the SLB was monitored by NR with datasets collected continuously until an equilibrium interaction between the protein and the SLB was verified by no further changes in the data being observed against time. At this point, a final equilibrium dataset was collected, and, then, the excess protein was flushed from the cell and the structure of the surface protein-lipid complex was examined by NR under three solution isotopic contrast conditions (D_2_O, Si-MW, and H_2_O). It should be noted that no difference was found in any sample between the equilibrium Bax–bound data before and after flushing of the excess protein, suggesting the protein-lipid complexes formed at the sample surface were stable.

#### NR data analysis

NR data were analyzed using the RasCal software (A. Hughes, ISIS Spallation Neutron Source, Rutherford Appleton Laboratory) that uses optical matrix formalism (*55*) to fit layered models of the structure across bulk interfaces and allow for the simultaneous analysis of multiple NR datasets collected under different sample and isotopic contrast conditions to be fully or partially constrained to the same surface structure in terms of thickness profile but vary in terms of neutron SLD.

The NR experiments were conducted in four stages: (i) the analysis of the bare silicon surfaces, (ii) the analysis of the SLB before Bax interaction, (iii) analysis of the kinetics while Bax was interacting with the membrane using a single solution isotopic contrast, and (iv) the analysis of the equilibrium protein-lipid surface complex. For details of NR analysis, see the Supplementary Text.

## Supplementary Materials

### This PDF file includes:

Supplementary Text Figs. S1 to S8 Tables S1 to S3 References

## Notes

View/request a protocol for this paper from *Bio-protocol*.
