# Supplementary material for: Creation of distinctive Bax-lipid complexes at mitochondrial membrane surfaces drives pore formation to initiate apoptosis
Source: Sci Adv. 2023 Jun 2;9(22):eadg7940. doi: 10.1126/sciadv.adg7940 (PMC10413641; doi:10.1126/sciadv.adg7940)
Supplement: Supplementary file 1 — Supplementary Text Figs. S1 to S8 Tables S1 to S3 References [file sciadv.adg7940_sm.pdf]

Science Advances
AAAS

Supplementary Materials for

Creation of distinctive Bax-lipid complexes at mitochondrial membrane surfaces drives pore formation to initiate apoptosis

Luke A. Clifton *et al.*

Corresponding author: Luke A. Clifton, luke.clifton@stfc.ac.uk; Gerhard Gröbner, gerhard.grobner@chem.umu.se

Sci. Adv. **9**, eadg7940 (2023)
DOI: 10.1126/sciadv.adg7940

This PDF file includes:

Supplementary Text
Figs. S1 to S8
Tables S1 to S3
References

Supplementary Text

Extended Methods

Analysis of NR experiments

Data was fitted using RasCal's custom model option where a bespoke script is used to describe the relationship between the fitted experimental parameters, the interfacial layers, the layer structure and the resulting SLD profile which is used to calculate the model reflectivity data sets which were fitted the experimental data. Models were constructed which constrained steps 1-4 of the experiment process to the same substrate (silicon and silicon-dioxide) structure.

For the majority of samples the membrane structure prior to Bax interaction was fitted as a four layer model, which, moving from the substrate to the bulk solution, was a thin SiO_2 layer, the inner bilayer head-groups, bilayer tails and the outer bilayer head-groups. The expected molecular volume and scattering length of the bilayer head/tails was determined from the molecular components of each lipid and the ratio of POPC and cardiolipin in the bilayers. The molar ratio of head and tail components in the bilayer was maintained by fitting an average lipid area per molecule for the bilayer where the thickness of the layers is defined by:

$$Layer\ thickness\ [\text{Å}] = \frac{Component\ Molecular\ Volume\ [\text{Å}^3]}{Area\ per\ molecule\ [\text{Å}^2]} \qquad \text{Eq.1}$$

And the SLD is defined by:

$$SLD\ [\text{Å}^{-2}] = \frac{\Sigma b\ [\text{Å}]}{Component\ Molecular\ Volume\ [\text{Å}^3]} \qquad \text{Eq. 2}$$

To allow for the difference in hydration between the hydrophobic head-groups and the hydrophilic tails of the bilayer two water parameters were fitted, membrane defect water which is hydration found both in the head-groups and tails due to defect in the SLB in the plane of the surface and head-group bound waters. In the case of all SLBs described in this study there was very little defect water detected prior to the interaction of Bax with the coverage of the SLBs on the substrate surface always approaching 100%.

Upon the equilibrium binding of Bax a number of increasingly complex models were used to satisfactorily fit the experimental data to a layer model. Ultimately, the model which suitably resolved all the features present in the experimental data sets was a seven layer model of a disrupted SLB and a protein-lipid complex on the bilayer surface. The initial four layers of the structure were the same as for the bilayer prior to the proteins interaction but with a large increase in the protein and water content and decrease in the lipid content. The three additional layers were found to be composed of a mixture of protein and lipid with a protein only layer bound to the SLB outer head-group region, a complex protein-lipid layer next to this and a protein only layer bound to this adjacent to the bulk solution.

Data sets which were collected against time during the interaction of Bax with the SLBs were fitted as a weighted average of the structures before and after the equilibrium interaction of Bax with the model mitochondrial surface.

NR Error Estimation and Plotting: Bayesian inference of the ambiguity of the resolved structures from NR model-to-data fitting was undertaken using MCMCStat (https://mjlaine.github.io/mcmcstat) Delayed-Rejection Adaptive Metropolis algorithms (DRAM) (56) Monte-Carlo-Markov Chain (MCMC) routines to refit the data using a user defined number of steps. To fit data using this approach, the likelihood function is defined in terms of the Chi-squared goodness of fit criteria, as shown previously (57). The parameter uncertainties were then

determined from the posterior distributions as the shortest percentile confidence interval (58) from each (in this case 65%) and the uncertainties on the reflectivity's and SLD's were generated by randomly sampling (in this case 1000 samples) from the Markov chains, calculating reflectivity's and SLD's for each set of samples, and taking the relevant percentile across all the resampled reflectivity or SLD curves at each point in Q_z (or distance) to represent the uncertainties on the fits. The chain samples are also used to generate the line shading used to denote the ambiguity of the structure across the interface in the volume fraction profiles. Best fit lines are the mean of the reflectivity and SLD uncertainties and are shown as a darker line in figures.

Volume fraction profiles detailing the distribution of components across the solid/liquid interface before and after the equilibrium interaction of Bax were produced using a bespoke script. MCMC Bayesian error estimation results and the relationship between the fitting parameters and interfacial structure in the RasCal custom model were used to determine the distribution of each structural component in the volume fraction vs. distance profile. The volume fraction of an individual component was calculated in 1 Å increments across the solid/liquid interface (the silicon/silicon dioxide interface set as zero). The mean, lower, and upper 65% confidence interval bounds of each component distribution were determined for every 1 Å segment using the MCMC error estimation results or derived parameters; these confidence intervals were then used to produce a line width error region above and below the mean values. The water distribution was calculated as the remaining unoccupied volume for each 1 Å slice and summed across the interface with the appropriate error propagation.

Attenuated Total Reflection Fourier Transform Infra-Red Spectroscopy (ATR-FTIR): Trapezoidal silicon substrates for attenuated total reflection infrared spectroscopy were obtained from Crystran (Poole, UK). These substrates were made to fit into a Specac (Orpington, UK) liquids ATR accessory which was, itself, fitted into the sample cavity Thermo-Fisher iS50 Infra-red spectrometer (Waltham, MA, USA). The substrates have four polished faces (to ~6 Å root mean squared roughness) the largest being a 72 mm × 10 mm face which was used as the sample surface. The IR beam enters the substrate through a polished face at 45° relative to the sample surface, total internal reflectance of the IR beam inside the substrate gives rise to six evanescent waves on the sample surface. IR spectra were collected at a resolution of 4 cm^{-1}.

To reduce the influence of water vapour on the IR spectra the iS50 instrument and ATR mirror assembly accessory was continuously purged with dry air from a Peak scientific (Glasgow, UK) CO_2 and water removing air purge. The Specac liquid ATR cell was modified to fit Omni-fit tubing which was connected to a syringe pump (AL1000-220, World Precision Instruments, Hitchin, UK). The experimental D_2O buffer solution (20 mM Tris pD 7.2 150 mM NaCl 2 mM $CaCl_2$) was injected into the ATR flow cell which was then heated to 37±1°C using a water bath (Julabo, Seelbach Germany). D_2O solutions were used for all ATR-FTIR measurements as the D_2O bending mode is lower (1215 cm-1) compared to H_2O (1645 cm-1) meaning limited contamination of protein amide I region by spectral bands from water (59). After buffer flushing a background spectra was collected. Spectra were then collected monitoring the removal of water vapor from the spectrometer and mirror assembly until a steady state was reached. A 2nd background measurement was then collected before deposition of the SLB.

SLB deposition was conducted using the same methodology as described for NR measurements (osmotic shock) and monitored through the appearance of CH_3 asymmetric, CH_2 asymmetric and CH_2 symmetric stretches from the lipid tails at ~2950 cm^{-1}, 2920 cm^{-1} and 2850 cm^{-1} respectively and the appearance of a lipid carbonyl stretch

at 1730 cm^{-1}. Once the SLB was formed BAX was injected into the solid/liquid flow cell its accumulation at the near surface region was monitored through the appearance of a protein Amide I peak.

Spectral deconvolution of the protein Amide I band was used to assess the major secondary structural components of Bax when bound to SLB models of the mitochondrial outer membrane by resolving the individual peaks which make up this broad band. This was done using the Fourier-Self deconvolution tool in the OMNIC software (version 9, Waltham, MA, USA) used to control the iS50 instrument. Deconvolution took place over a spectral range of ~1600 – ~1700 cm-1 using a deconvolution bandwidth of 32 cm^{-1} and spectral enhancement of 2.1. Assignment of the resolved secondary structural components was conducted by comparing the predominant resolved overlapping band which made up the Bax Amide I band to spectral values of differing protein secondary structural components in D_2O solution from literature (*59*).

Fig. S1.

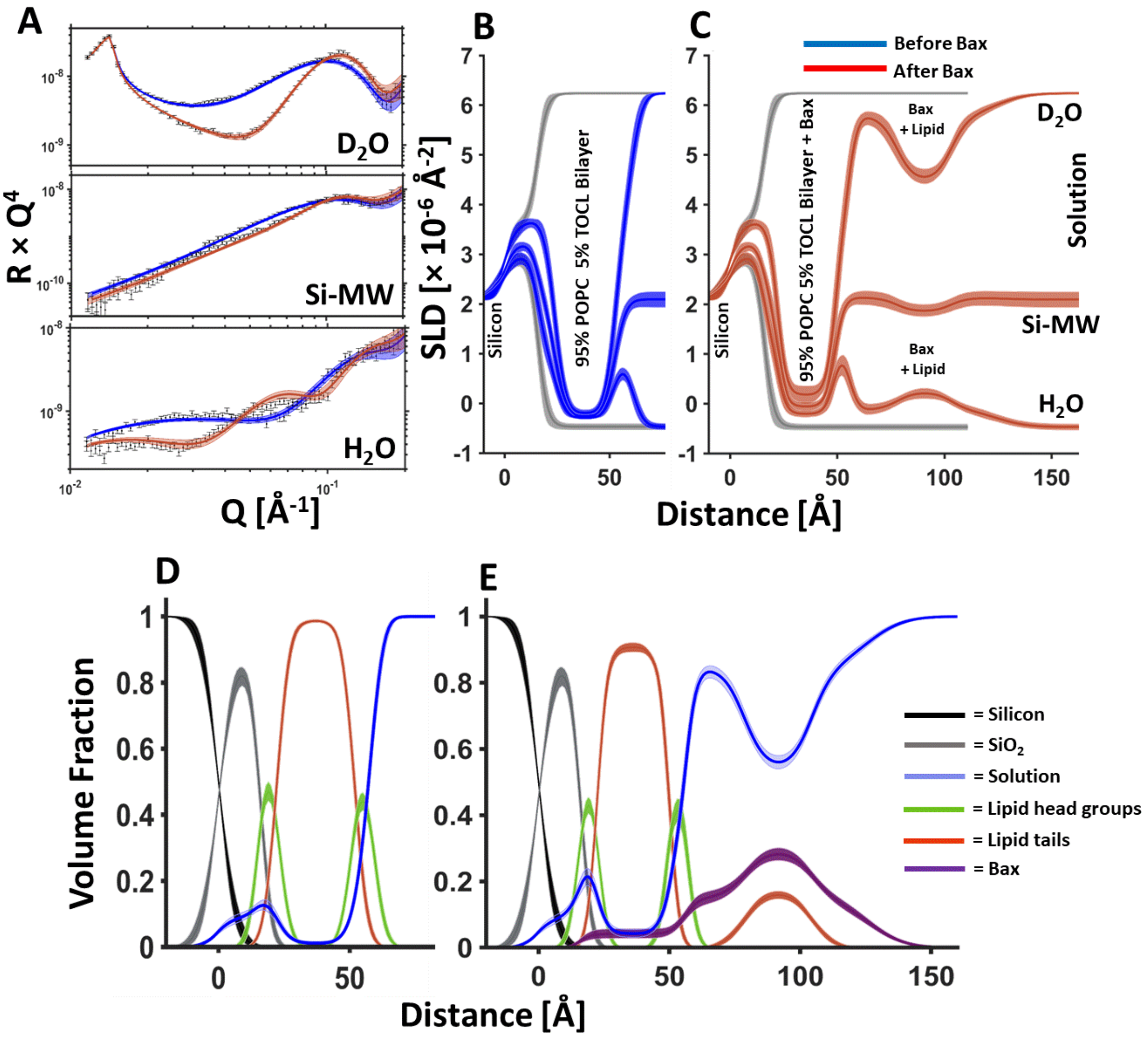

Fig. S1. NR data showing the structure at a silicon/water interface containing a POPC-5 mol% TOCL SLB before and after the interaction of Bax. NR data (error bars) and model data fits (lines) from a POPC:TOCL SLB before (blue) and after (red) the interaction of natural abundance hydrogen (h-)Bax are shown in three differing solution isotopic contrast conditions being D_2O, Si-MW and H_2O (A) buffer solutions. The scattering length density (SLD) profiles the model fits represent are shown for the surface structure before (B) and after the h-Bax interaction (C). The component volume fraction profiles at the Si/water interface before (D) and after (E) the h-Bax interaction determined from the NR fits are shown. Here the silicon distribution is given in black, silicon dioxide is grey, water is blue, lipid head-groups are green, lipid tails are red and the Bax protein distribution is in purple. Line widths in the NR data fits represent the 65% confidence interval of the range of acceptable fits determined from MCMC error analysis and the line widths on the SLD and volume fraction profiles represent the ambiguity in the resolved interfacial structure determined from these.

Fig. S2.

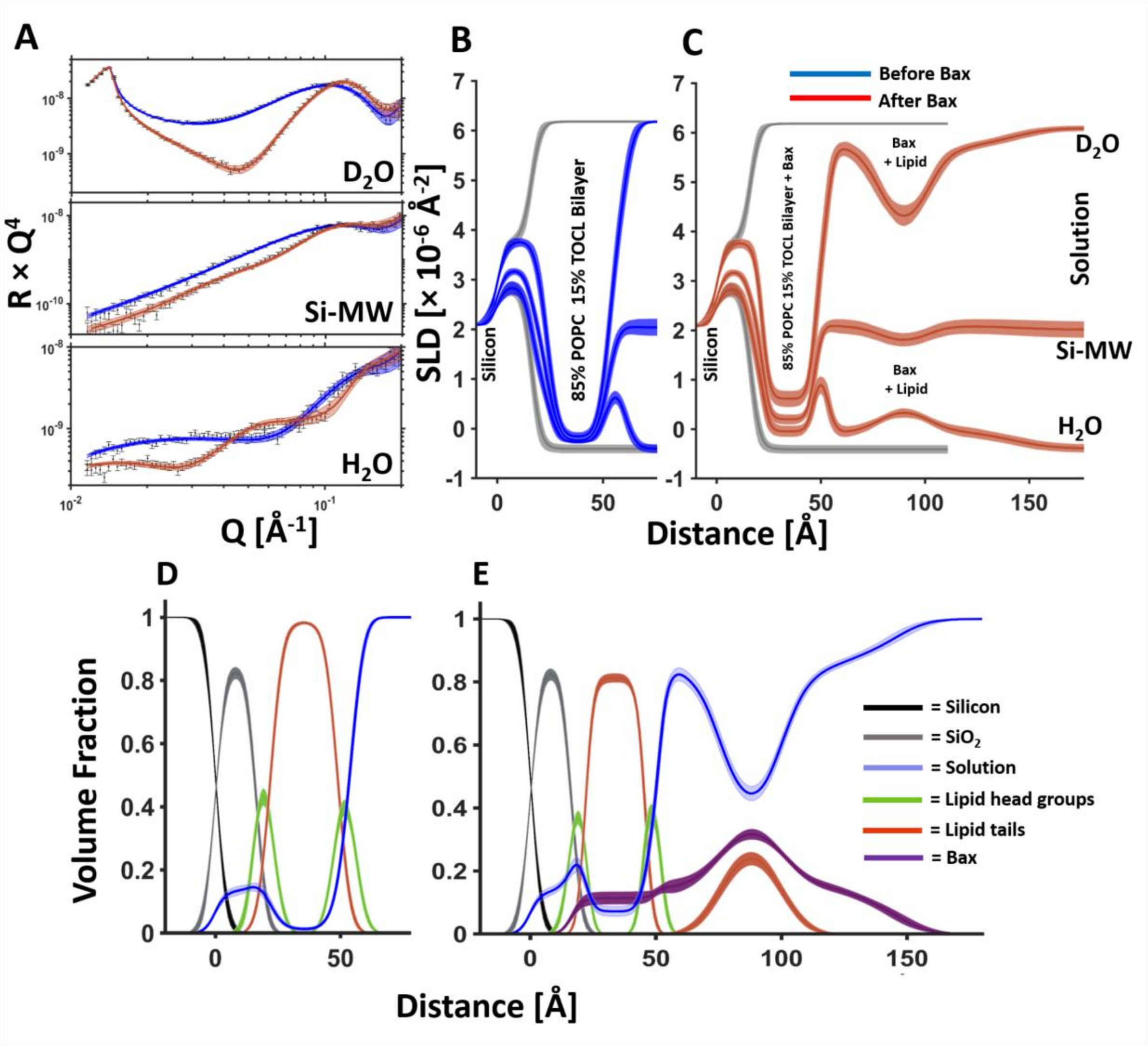

Fig. S2. NR data showing the structure at a silicon/water interface containing a POPC-15 mol% TOCL SLB before and after the interaction of Bax. NR data (error bars) and model data fits (lines) from a POPC:TOCL SLB before (blue) and after (red) the interaction of natural abundance hydrogen (h-)Bax are shown in three differing solution isotopic contrast conditions being D_2O, Si-MW and H_2O (A) buffer solutions. The scattering length density (SLD) profiles the model fits represent are shown for the surface structure before (B) and after the h-Bax interaction (C). The component volume fraction profiles at the Si/water interface before (D) and after (E) the h-Bax interaction determined from the NR fits are shown. Here the silicon distribution is given in black, silicon dioxide is grey, water is blue, lipid head-groups are green, lipid tails are red and the Bax protein distribution is in purple. Line widths in the NR data fits represent the 65% confidence interval of the range of acceptable fits determined from MCMC error analysis and the line widths on the SLD and volume fraction profiles represent the ambiguity in the resolved interfacial structure determined from these.

Fig. S3.

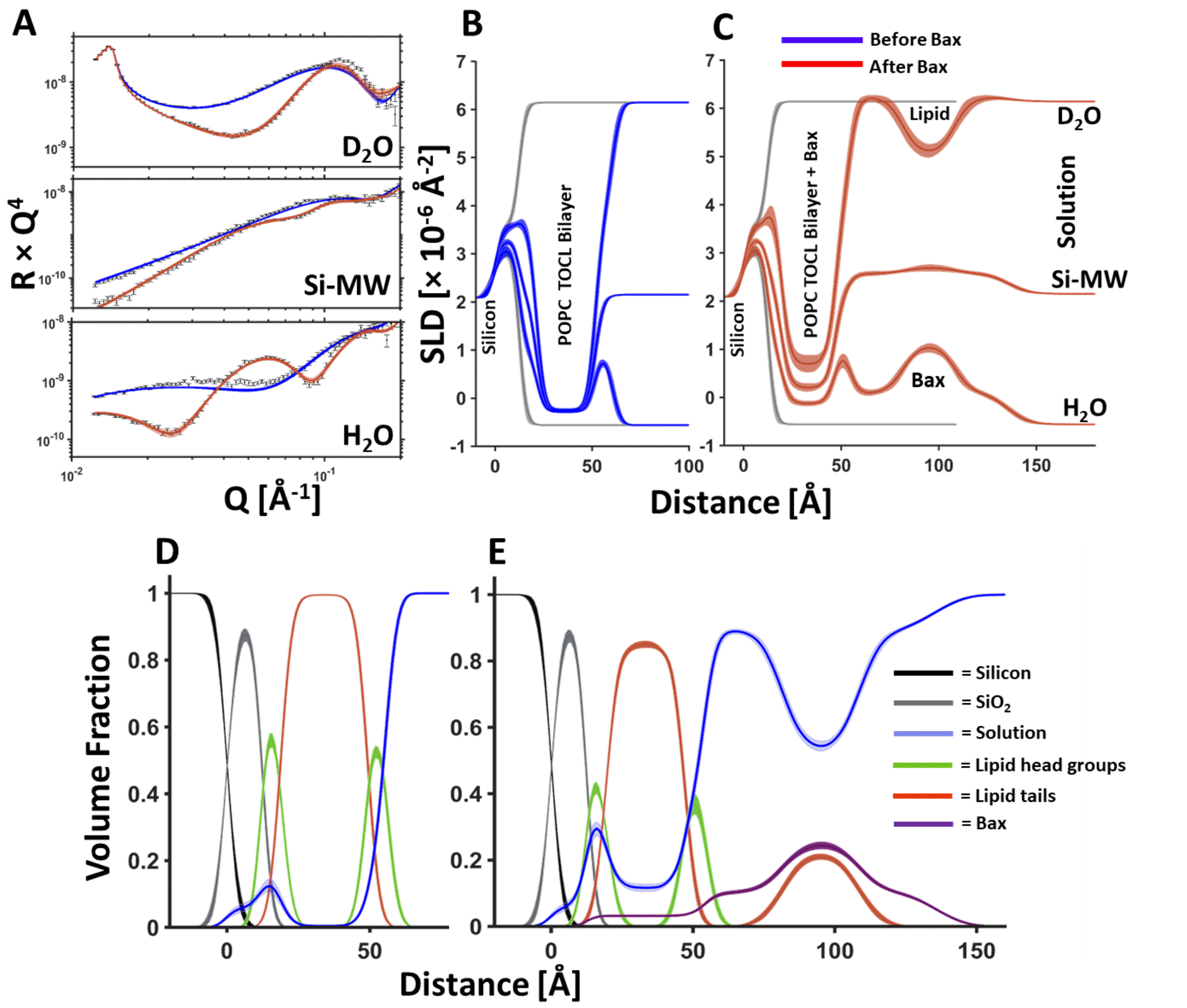

Fig. S3. NR data showing the structure at a silicon/water interface containing a POPC-10 mol% TOCL SLB before and after the interaction of deuterated (d-)Bax. NR data (error bars) and model data fits (lines) from a POPC:TOCL SLB before (blue) and after (red) the interaction of natural abundance hydrogen (d-)Bax are shown in three differing solution isotopic contrast conditions being D_2O, Si-MW and H_2O (A) buffer solutions. The scattering length density (SLD) profiles the model fits represent are shown for the surface structure before (B) and after the d-Bax interaction (C). The component volume fraction profiles at the Si/water interface before (D) and after (E) the d-Bax interaction determined from the NR fits are shown. Here the silicon distribution is given in black, silicon dioxide is grey, water is blue, lipid head-groups are green, lipid tails are red and the Bax protein distribution is in purple. Line widths in the NR data fits represent the 65% confidence interval of the range of acceptable fits determined from MCMC error analysis and the line widths on the SLD and volume fraction profiles represent the ambiguity in the resolved interfacial structure determined from these. Note how by using d-Bax for the interaction (which has a similar SLD to D_2O) the lipid component of the Bax/lipid membrane MOM surface clusters was independently resolved in the D_2O solution isotopic contrast (E).

Fig. S4.

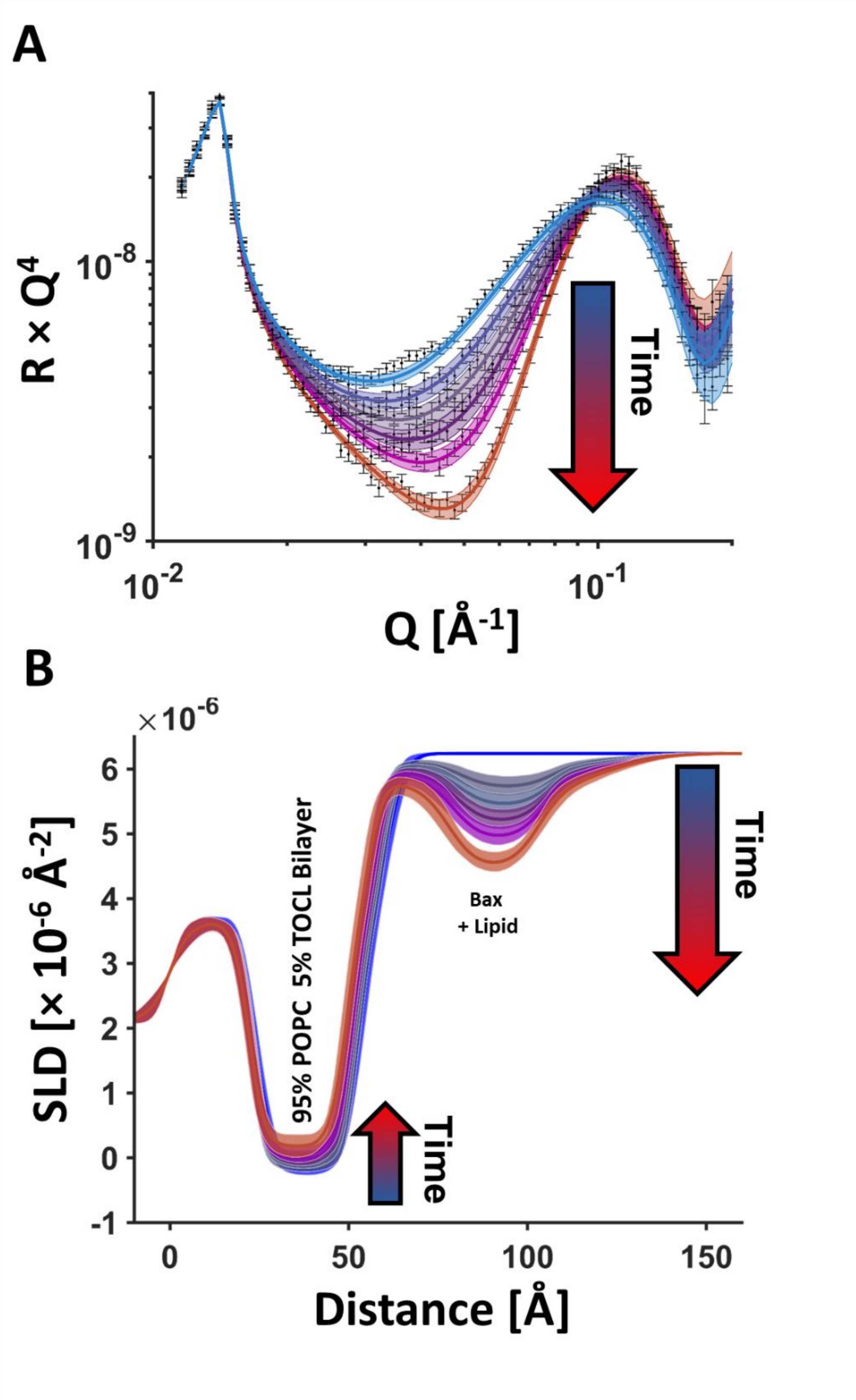

Fig. S4. Time resolved NR data (error bars) and model data fits (lines) from a POPC-5 mol% TOCL SLB. Time dependence of NR data during the interaction of (h-)Bax in a D_2O buffer solution (A); and the changes in the scattering length density of the surface assemblage against time (B, depicted from blue to red). Note how the increasing SLD of the bilayer, depicting lipid loss, is commensurate with decreasing SLD of the Bax/Lipid surface complex, which depicting increasing complex coverage. Lines widths on the fits/SLD profiles depict the ambiguity in the resolved structure from MCMC error estimation.

Fig. S5.

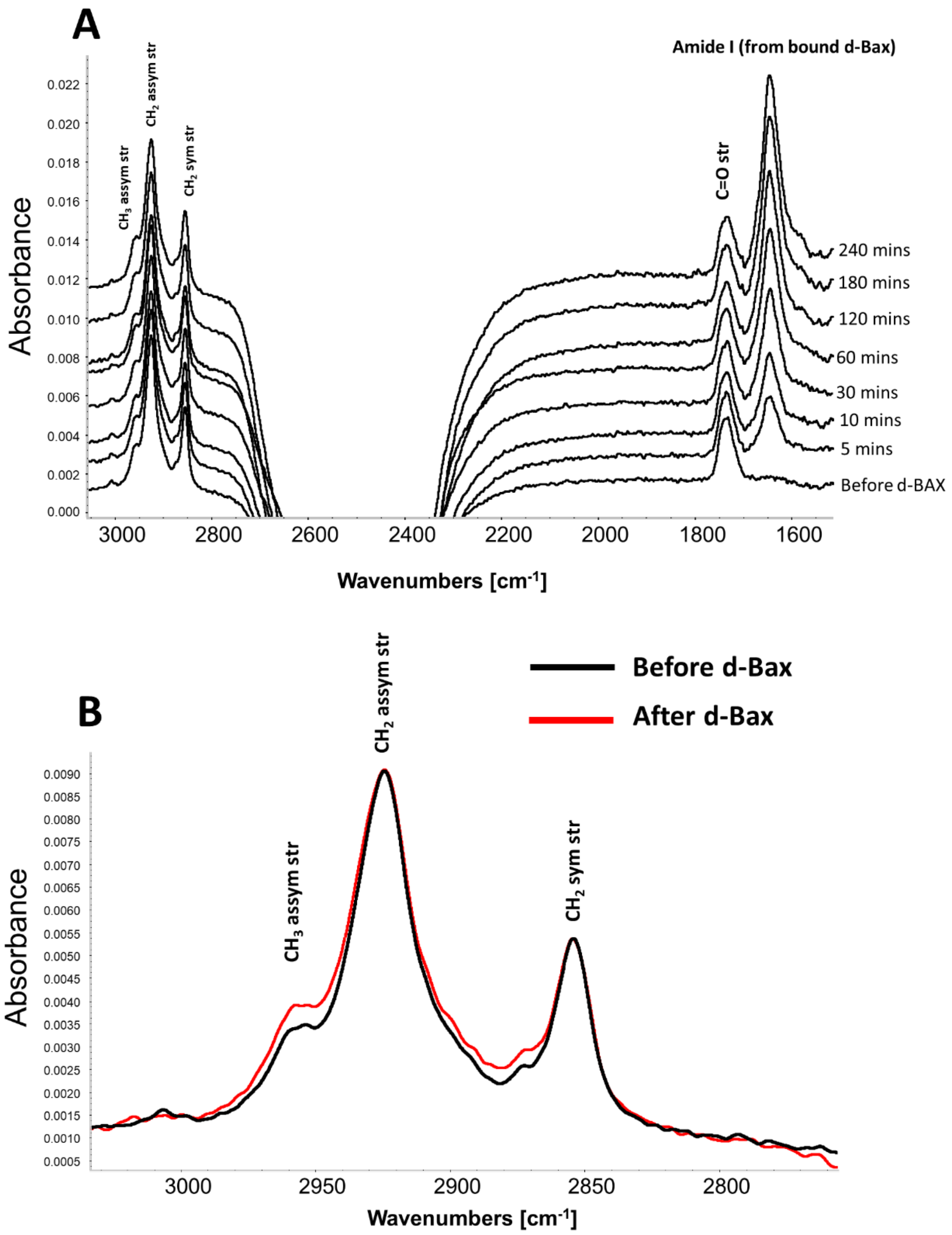

Fig. S5. ATR-FTIR spectra obtained during the interaction of d-Bax with a POPC-10 mol% TOCL SLB at the silicon/D_2O buffer interface. Time dependence of the main spectral features (A); A comparison (B) of the CH_2 stretch region of the IR spectra showing the differences in the bands in this region before (back line) and after equilibrium d-Bax binding (red line).

Fig. S6.

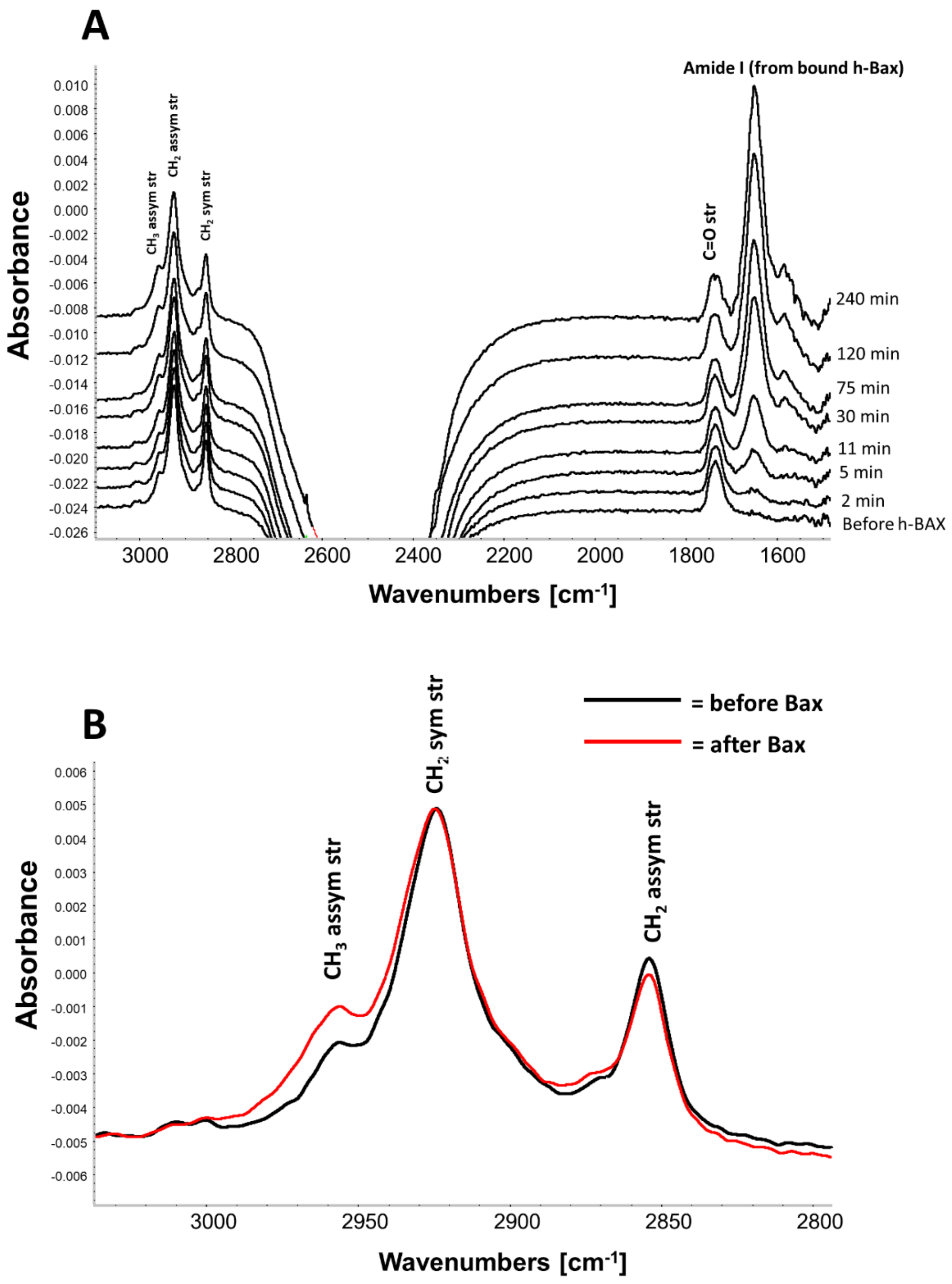

Fig. S6. ATR-FTIR spectra obtained during the interaction of h-Bax with a POPC-10 mol% TOCL SLB at the silicon/D_2O buffer interface. Time-dependence of the main spectral features (A); A comparison (B) of the CH_2 stretch region of the IR spectra showing the differences in the bands in this region before (back line) and after equilibrium h-Bax binding (red line).

Fig. S7.

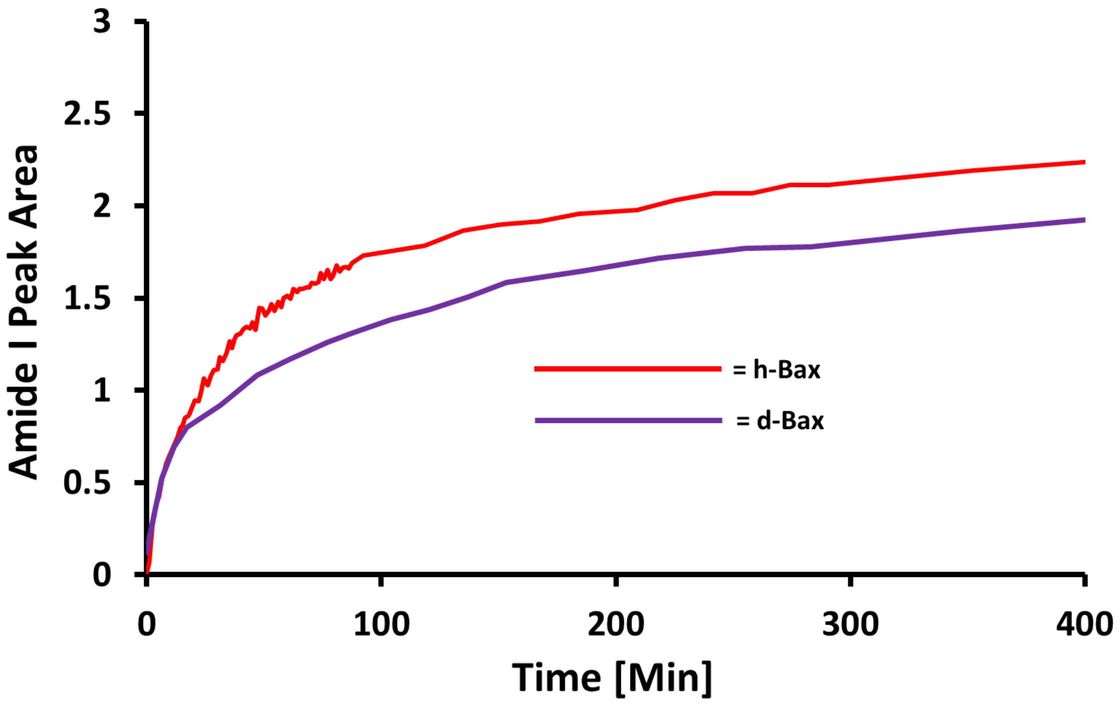

Fig. S7. A comparison between the change in Amide I peak area vs. time for h-Bax (red) and d-Bax (purple) during the interaction of these proteins with POPC-10 mol% TOCL SLBs examined by ATR-FTIR. Examples of the spectra used to generate this plot are shown in Figure S5 for d-Bax and S6 for h-Bax. Peak areas were normalized to the CH_2 asymmetric stretch from the bilayer lipid tails.

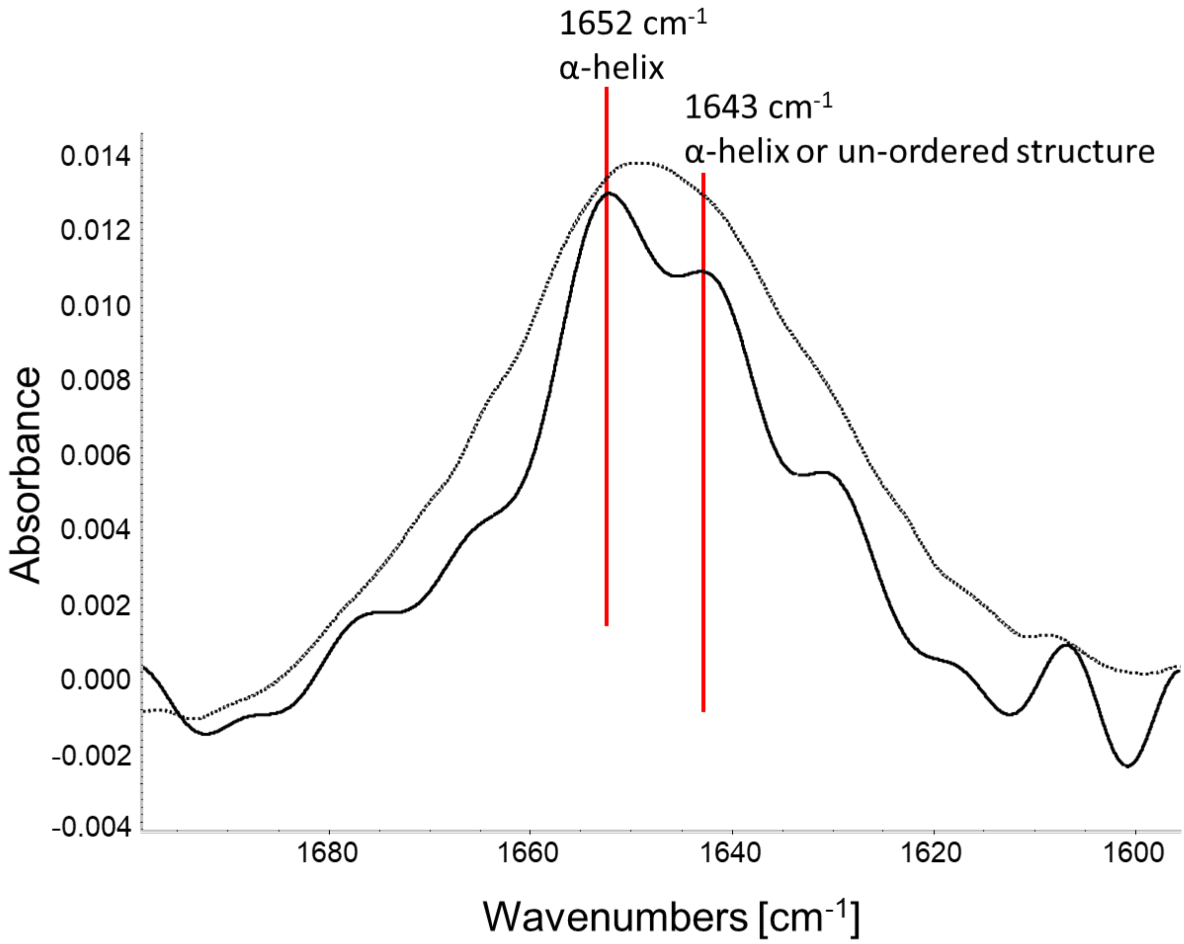

Fig S8. Protein Amide I region (dotted line) and a spectral deconvolution of this band (solid line) from ATR-FTIR spectra of h-Bax with POPC-10 mol% TOCL SLB at the silicon/D_2O buffer interface (see Fig. S6). Results of spectral deconvolution suggest the major secondary component of the protein upon interaction with the model MOM membrane is α-helix (peak at 1652 cm^{-1}) with a second major component (band at 1643 cm^{-1}) which could be assigned as α-helix or unordered structure based on previous literature (*59*).

Table S1. The resolved structural components before and after the interaction of (h-)Bax with SLB composed of POPC- 5 mol% TOCL.*

	Average Lipid Area per Molecule [Å^2]	Tails Thickness [Å]	Tail Composition [vol%]	Head Group Thickness [Å]	Outer head-group Composition [vol%]	Membrane Surface Bax/Lipid Complex Thickness [Å]	Bax Surface Layer Composition [vol%]
**POPC:TOCL (95:5 mol%)**	64.8 Å^2 (62.4 Å^2, 64.2 Å^2)	30.3 Å (30.0 Å, 30.6 Å)	Lipid 99% (98%, 100%)<br><br>Solution 1% (0%, 2%)	6.2 Å (6.0 Å, 6.4 Å)	Head-group 83% (80%, 85%)<br><br>Water 17% (15%, 20%)	-	-
**POPC:TOCL (95:5 mol%) + h-Bax**	70.1 Å^2 (69.0 Å^2, 71.3 Å^2)	27.3 Å (26.8 Å, 27.7 Å)	Lipid 91% (90%, 92%)<br><br>Protein 4% (3%, 5%)<br><br>Solution 4% (3%, 5%)	6.6 Å (6.2 Å, 7.0 Å)	Lipid 66% (63%, 70%)<br><br>Protein 4% (3%, 5%)<br><br>Solution 29% (26%, 32%)	**1**, 22.6 Å (21.4 Å, 23.6 Å)<br><br>**2**, 24.8 Å (23.2 Å, 26.0 Å)<br><br>**3**, , 22.6 Å (21.4 Å, 23.6 Å)<br><br>**Total: 70.4 Å (69.1 Å, 71.7 Å)**	**Protein Lipid Distribution Composition:**<br>1, Protein 15% (14%, 16%)<br>Water 85 % (84%, 86%)<br><br>2, Protein 29 % (27%, 32%)<br>Lipid 15% (16%, 18%)<br>Solution 55% (52%, 57%)<br><br>3, Protein 15% (14%, 16%)<br>Water 85 % (84%, 86%)

* Values in brackets represent the 65% confidence intervals determined from MCMC resampling of the experimental data fits.

Table S2.

Table S2. The resolved structural components before and after the interaction of (h-)Bax with SLB composed of POPC- 15 mol% TOCL .*

	Average Lipid Area per Molecule [$Å^2$]	Tails Thickness [Å]	Tails Composition [vol%]	Head Group Thickness [Å]	Outer head-group Composition [vol%]	Membrane Surface Bax/Lipid Complex Thickness [Å]	Bax Surface Layer Composition [vol%]
**POPC:TOCL (85:15 mol%)**	71.4 $Å^2$ (70.7 $Å^2$, 72.2 $Å^2$)	26.9 Å (26.6 Å, 27.2 Å)	Lipid 99% (98%, 100%)<br><br>Solution 1% (0%, 2%)	5.6 Å (5.4 Å ,5.8 Å)	Head-group 82% (79%, 85%)<br>Water 18% (15%, 21%)	-	-
**POPC:TOCL (85:15 mol%) + h-BAX**	81.1 $Å^2$ (79.8 $Å^2$, 82.4 $Å^2$)	23.6 Å (23.3 Å, 24.0 Å)	Lipid 81% (80%, 82%)<br><br>Protein 11% (10%, 14%)<br><br>Solution 8% (6%, 10%)	5.7 Å (5.4 Å, 6.0 Å)	Lipid 60% (57%, 64%)<br><br>Protein 11% (10%, 14%)<br><br>Solution 29% (25%, 32%)	**1**, 24.5 Å (22.6 Å, 25.9 Å)<br><br>**2**, 23.7 Å (21.0 Å, 27.0 Å)<br><br>**3**, , 43.0 Å (38.7 Å, 47.3 Å)<br><br>**Total: 91.7 Å (87.3 Å, 96.3 Å)**	**Protein Lipid Distribution Composition:**<br>1, Protein 14% (11%, 17%)<br>Water 86 % (83%, 89%)<br><br>2, Protein 35 % (32%, 39%)<br>Lipid 19% (17%, 21%)<br>Solution 46% (41%, 50%)<br><br>3, Protein 15% (13%, 17%)<br>Water 85 % (83%, 87%)

* Values in brackets represent the 65% confidence intervals determined from MCMC resampling of the experimental data fits.

Table S3

Table S3. The resolved structural components before and after the interaction of (d-)Bax with SLB composed of 9:1 (mol/mol) POPC:TOCL.*

	**Average Lipid Area per Molecule [Å^2]**	**Tails Thickness [Å]**	**Tails Composition [vol%]**	**Head Group Thickness [Å]**	**Outer head-group Composition [vol%]**	**Membrane Surface Bax/Lipid Complex Thickness [Å]**	**Bax Surface Layer Composition [vol%]**
**POPC:TOCL (90:10 mol%)**	63.1 Å^2 (62.7 Å^2, 63.6 Å^2)	30.4 Å (30.2 Å, 30.6 Å)	Lipid 100% (99%, 100%)<br><br>Solution 0% (0%, 1%)	6.3 Å (6.1 Å ,6.5 Å)	Head-group 84% (81%, 86%)<br><br>Water 16% (14%, 19%)	-	-
**POPC:TOCL (90:10 mol%) + d-BAX**	69.4 Å^2 (68.4 Å^2, 70.4 Å^2)	27.6 Å (27.2 Å, 28.0 Å)	Lipid 85% (81%, 85%)<br><br>Protein 3% (2%, 4%)<br><br>Solution 12% (11%, 13%)	7.0 Å (6.7 Å, 7.2 Å)	Lipid 59% (57%, 61%)<br><br>Protein 3% (2%, 4%)<br><br>Solution 38% (36%, 40%)	**1**, 28.0 Å (26.9 Å, 29.0 Å)<br><br>**2**, 25.9 Å (24.2 Å, 27.9 Å)<br><br>**3**, 28.0 Å (26.9 Å, 29.0 Å)<br><br>**Total: 83.1 Å (82.0 Å, 84.2 Å)**	**Protein Lipid Distribution Composition:**<br>1, Protein 10% (9%, 11%)<br>Water 90% (89%, 91%)<br><br>2, Protein 26% (25%, 27%)<br>Lipid 22% (21%, 23%)<br>Solution 51% (49%, 53%)<br><br>3, Protein 10% (9%, 11%)<br>Water 90% (89%, 91%)

* Values in brackets represent the 65% confidence intervals determined from MCMC resampling of the experimental data fits

REFERENCES AND NOTES

1. J. C. Martinou, R. J. Youle, Mitochondria in apoptosis: Bcl-2 family members and mitochondrial dynamics. *Dev. Cell* **21**, 92–101 (2011).

2. J. Kale, E. J. Osterlund, D. W. Andrews, BCL-2 family proteins: Changing partners in the dance towards death. *Cell Death Differ.* **25**, 65–80 (2018).

3. K. J. Campbell, S. W. G. Tait, Targeting BCL-2 regulated apoptosis in cancer. *Open Biol.* **8**, 180002 (2018).

4. S. Grabow, A. J. Kueh, F. Ke, H. K. Vanyai, B. N. Sheikh, M. A. Dengler, W. Chiang, S. Eccles, I. M. Smyth, L. K. Jones, F. J. de Sauvage, M. Scott, L. Whitehead, A. K. Voss, A. Strasser, Subtle changes in the levels of BCL-2 proteins cause severe craniofacial abnormalities. *Cell Rep.* **24**, 3285–3295.e4 (2018).

5. R. B. Hill, K. R. MacKenzie, M. C. Harwig, The tail-end is only the beginning: NMR study reveals a membrane-bound state of BCL-XL. *J. Mol. Biol.* **427**, 2257–2261 (2015).

6. G. C. Brito, W. Schormann, S. K. Gidda, R. T. Mullen, D. W. Andrews, Genome-wide analysis of *Homo sapiens*, *Arabidopsis thaliana*, and *Saccharomyces cerevisiae* reveals novel attributes of tail-anchored membrane proteins. *BMC Genomics* **20**, 835 (2019).

7. A. U. Mushtaq, J. Aden, L. A. Clifton, H. Wacklin-Knecht, M. Campana, A. P. G. Dingeldein, C. Persson, T. Sparrman, G. Grobner, Neutron reflectometry and NMR spectroscopy of full-length Bcl-2 protein reveal its membrane localization and conformation. *Commun. Biol.* **4**, 507 (2021).

8. M. Suzuki, R. J. Youle, N. Tjandra, Structure of Bax. *Cell* **103**, 645–654 (2000).

9. S. Bleicken, G. Hofhaus, B. Ugarte-Uribe, R. Schroder, A. J. Garcia-Saez, cBid, Bax and Bcl-xL exhibit opposite membrane remodeling activities. *Cell Death Dis.* **7**, e2121 (2016).

10. A. Shamas-Din, S. Bindner, X. K. Chi, B. Leber, D. W. Andrews, C. Fradin, Distinct lipid effects on tBid and Bim activation of membrane permeabilization by pro-apoptotic Bax. *Biochem. J.* **467**, 495–505 (2015).

11. D. Westphal, G. Dewson, M. Menard, P. Frederick, S. Iyer, R. Bartolo, L. Gibson, P. E. Czabotar, B. J. Smith, J. M. Adams, R. M. Kluck, Apoptotic pore formation is associated with in-plane insertion of Bak or Bax central helices into the mitochondrial outer membrane. *Proc. Natl. Acad. Sci. U.S.A.* **111**, E4076–E4085 (2014).

12. Z. J. Hauseman, E. P. Harvey, C. E. Newman, T. E. Wales, J. C. Bucci, J. Mintseris, D. K. Schweppe, L. David, L. X. Fan, D. T. Cohen, H. D. Herce, R. Mourtada, Y. Ben-Nun, N. B. Bloch, S. B. Hansen, H. Wu, S. P. Gygi, J. R. Engen, L. D. Walensky, Homogeneous oligomers of pro-apoptotic BAX reveal structural determinants of mitochondrial membrane permeabilization. *Mol. Cell* **79**, 68–83.e7 (2020).

13. B. Schellenberg, P. B. Wang, J. A. Keeble, R. Rodriguez-Enriquez, S. Walker, T. W. Owens, F. Foster, J. Tanianis-Hughes, K. Brennan, C. H. Streuli, A. P. Gilmore, Bax exists in a dynamic equilibrium between the cytosol and mitochondria to control apoptotic priming. *Mol. Cell* **49**, 959–971 (2013).

14. F. Gonzalvez, E. Gottlieb, Cardiolipin: Setting the beat of apoptosis. *Apoptosis* **12**, 877–885 (2007).

15. Y. C. Lai, C. C. Li, T. C. Sung, C. W. Chang, Y. J. Lan, Y. W. Chiang, The role of cardiolipin in promoting the membrane pore-forming activity of BAX oligomers. *Biochim. Biophys. Acta Biomembr.* **1861**, 268–280 (2019).

16. V. Vasquez-Montes, M. V. Rodnin, A. Kyrychenko, A. S. Ladokhin, Lipids modulate the BH3-independent membrane targeting and activation of BAX and Bcl-xL. *Proc. Natl. Acad. Sci. U.S.A.* **118**, e2025834118 (2021).

17. R. Salvador-Gallego, M. Mund, K. Cosentino, J. Schneider, J. Unsay, U. Schraermeyer, J. Engelhardt, J. Ries, A. J. Garcia-Saez, Bax assembly into rings and arcs in apoptotic mitochondria is linked to membrane pores. *EMBO J.* **35**, 389–401 (2016).

18. A. Shamas-Din, D. Satsoura, O. Khan, W. Zhu, B. Leber, C. Fradin, D. W. Andrews, Multiple partners can kiss-and-run: Bax transfers between multiple membranes and permeabilizes those primed by tBid. *Cell Death Dis.* **5**, e1277 (2014).

19. M. Lidman, S. Pokorna, A. P. G. Dingeldein, T. Sparrman, M. Wallgren, R. Sachl, M. Hof, G. Grobner, The oxidized phospholipid PazePC promotes permeabilization of mitochondrial membranes by Bax. *Biochim. Biophys. Acta Biomembr.* **1858**, 1288–1297 (2016).

20. D. Ardail, J. P. Privat, M. Egret-Charlier, C. Levrat, F. Lerme, P. Louisot, Mitochondrial contact sites. Lipid composition and dynamics. *J. Biol. Chem.* **265**, 18797–18802 (1990).

21. S. E. Horvath, G. Daum, Lipids of mitochondria. *Prog. Lipid Res.* **52**, 590–614 (2013).

22. M. Z. Zhang, J. Zheng, R. Nussinov, B. Y. Ma, Release of cytochrome C from Bax pores at the mitochondrial membrane. *Sci. Rep.* **7**, 2635 (2017).

23. S. Bleicken, T. E. Assafa, C. Stegmueller, A. Wittig, A. J. Garcia-Saez, E. Bordignon, Topology of active, membrane-embedded Bax in the context of a toroidal pore. *Cell Death Differ.* **25**, 1717–1731 (2018).

24. K. Cosentino, V. Hertlein, A. Jenner, T. Dellmann, M. Gojkovic, A. Pena-Blanco, S. Dadsena, N. Wajngarten, J. S. H. Danial, J. V. Thevathasan, M. Mund, J. Ries, A. J. Garcia-Saez, The interplay between BAX and BAK tunes apoptotic pore growth to control mitochondrial-DNA-mediated inflammation. *Mol. Cell* **82**, 933–949.e9 (2022).

25. N. R. Ader, P. C. Hoffmann, I. Ganeva, A. C. Borgeaud, C. X. Wang, R. J. Youle, W. Kukulski, Molecular and topological reorganizations in mitochondrial architecture interplay during Bax-mediated steps of apoptosis. *eLife* **8**, e40712 (2019).

26. L. A. Clifton, Unravelling the structural complexity of protein-lipid interactions with neutron reflectometry. *Biochem. Soc. Trans.* **49**, 1537–1546 (2021).

27. L. A. Clifton, R. A. Campbell, F. Sebastiani, J. Campos-Teran, J. F. Gonzalez-Martinez, S. Bjorklund, J. Sotres, M. Cardenas, Design and use of model membranes to study biomolecular interactions using complementary surface-sensitive techniques. *Adv. Colloid Interface. Sci.* **277**, 102118 (2020).

28. N. Paracini, L. A. Clifton, M. W. A. Skoda, J. H. Lakey, Liquid crystalline bacterial outer membranes are critical for antibiotic susceptibility. *Proc. Natl. Acad. Sci. U.S.A.* **115**, E7587–E7594 (2018).

29. L. A. Clifton, S. A. Holt, A. V. Hughes, E. L. Daulton, W. Arunmanee, F. Heinrich, S. Khalid, D. Jefferies, T. R. Charlton, J. R. P. Webster, C. J. Kinane, J. H. Lakey, An accurate in vitro model of the *E. coli* envelope. *Angew. Chem. Int. Edit.* **54**, 11952–11955 (2015).

30. H. P. Wacklin, B. B. Bremec, M. Moulin, N. Rojko, M. Haertlein, T. Forsyth, G. Anderluh, R. S. Norton, Neutron reflection study of the interaction of the eukaryotic pore-forming actinoporin equinatoxin II with lipid membranes reveals intermediate states in pore formation. *Biochim. Biophys. Acta Biomembr.* **1858**, 640–652 (2016).

31. H. N. Gong, X. Z. Hu, M. R. Liao, K. Fa, D. Ciumac, L. A. Clifton, M. A. Sani, S. M. King, A. Maestro, F. Separovic, T. A. Waigh, H. Xu, A. J. McBain, J. R. Lu, Structural disruptions of the outer membranes of gram-negative bacteria by rationally designed amphiphilic antimicrobial peptides. *ACS Appl. Mater.* **13**, 16062–16074 (2021).

32. J. D. Gelles, J. E. Chipuk, Robust high-throughput kinetic analysis of apoptosis with real-time high-content live-cell imaging. *Cell Death Dis.* **7**, e2493 (2016).

33. S. Bleicken, M. Classen, P. V. Padmavathi, T. Ishikawa, K. Zeth, H. J. Steinhoff, E. Bordignon, Molecular details of Bax activation, oligomerization, and membrane insertion. *J. Biol. Chem.* **285**, 6636–6647 (2010).

34. F. J. Lv, F. Qi, Z. Zhang, M. R. Wen, J. Kale, A. Piai, L. Y. Du, S. Q. Wang, L. J. Zhou, Y. Q. Yang, B. Wu, Z. J. Liu, J. Rosario, J. Pogmore, J. J. Chou, D. W. Andrews, J. L. Lin, B. OuYang, An amphipathic Bax core dimer forms part of the apoptotic pore wall in the mitochondrial membrane. *EMBO J.* **40**, e106438 (2021).

35. T. Kuwana, N. H. Olson, W. B. Kiosses, B. Peters, D. D. Newmeyer, Pro-apoptotic Bax molecules densely populate the edges of membrane pores. *Sci. Rep.* **6**, 27299 (2016).

36. C. A. Lopez, M. F. Swift, X. P. Xu, D. Hanein, N. Volkmann, S. Gnanakaran, Biophysical characterization of a nanodisc with and without BAX: An integrative study using molecular dynamics simulations and cryo-EM. *Structure* **27**, 988–999.e4 (2019).

37. P. E. Czabotar, D. Westphal, G. Dewson, S. Ma, C. Hockings, W. D. Fairlie, E. F. Lee, S. G. Yao, A. Y. Robin, B. J. Smith, D. C. S. Huang, R. M. Kluck, J. M. Adams, P. M. Colman, Bax crystal structures reveal how BH3 domains activate bax and nucleate its oligomerization to induce apoptosis. *Cell* **152**, 519–531 (2013).

38. S. Qian, W. C. Wang, L. Yang, H. W. Huang, Structure of transmembrane pore induced by Bax-derived peptide: Evidence for lipidic pores. *Proc. Natl. Acad. Sci. U.S.A.* **105**, 17379–17383 (2008).

39. S. Lucken-Ardjomande, S. Montessuit, J. C. Martinou, Contributions to Bax insertion and oligomerization of lipids of the mitochondrial outer membrane. *Cell Death Differ.* **15**, 929–937 (2008).

40. A. Y. Robin, S. Iyer, R. W. Birkinshaw, J. Sandow, A. Wardak, C. S. Luo, M. Shi, A. I. Webb, P. E. Czabotar, R. M. Kluck, P. M. Colman, Ensemble properties of Bax determine its function. *Structure* **26**, 1346–1359.e5 (2018).

41. R. T. Uren, S. Iyer, R. M. Kluck, Pore formation by dimeric Bak and Bax: An unusual pore? *Philos. Trans. R. Soc. Lond. B Biol. Sci.* **372**, 20160218 (2017).

42. M. T. F. Telling, C. Neylon, L. Clifton, S. Howells, L. van Eijck, V. G. Sakai, The dynamic landscape in the multi-subunit protein, apoferritin, as probed by high energy resolution neutron spectroscopy. *Eur. Biophys. J. Biophys. Lett.* **40**, 156–156 (2011).

43. S. A. Tatulian, FTIR analysis of proteins and protein-membrane interactions. *Methods Mol. Biol.* **2003**, 281–325 (2019).

44. A. D. Cowan, N. A. Smith, J. J. Sandow, E. A. Kapp, Y. H. Rustam, J. M. Murphy, J. M. Brouwer, J. P. Bernardini, M. J. Roy, A. Z. Wardak, I. K. Tan, A. I. Webb, J. M. Gulbis, B. J. Smith, G. E. Reid, G. Dewson, P. M. Colman, P. E. Czabotar, BAK core dimers bind lipids and can be bridged by them. *Nat. Struct. Mol. Biol.* **27**, 1024–1031 (2020).

45. A. Poulaki, S. Giannouli, Mitochondrial lipids: From membrane organization to apoptotic facilitation. *Int. J. Mol. Sci.* **23**, 3738 (2022).

46. L. Grosse, C. A. Wurm, C. Bruser, D. Neumann, D. C. Jans, S. Jakobs, Bax assembles into large ring-like structures remodeling the mitochondrial outer membrane in apoptosis. *EMBO J.* **35**, 402–413 (2016).

47. K. Cosentino, A. J. Garcia-Saez, Bax and Bak pores: Are we closing the circle? *Trends Cell Biol.* **27**, 266–275 (2017).

48. S. Qian, V. K. Sharma, L. A. Clifton, Understanding the structure and dynamics of complex biomembrane interactions by neutron scattering techniques. *Langmuir* **36**, 15189–15211 (2020).

49. M. A. Sani, E. J. Dufourc, G. Grobner, How does the Bax-alpha1 targeting sequence interact with mitochondrial membranes? The role of cardiolipin. *Biochim. Biophys. Acta* **1788**, 623–631 (2009).

50. M. Crimi, M. D. Esposti, Apoptosis-induced changes in mitochondrial lipids. *Biochim. Biophys. Acta* **1813**, 551–557 (2011).

51. A. P. G. Dingeldein, M. J. Lindberg, J. Aden, X. Zhong, R. Stoll, G. Grobner, Bax to the future – A novel, high-yielding approach for purification and expression of full-length Bax protein for structural studies. *Protein Expr. Purif.* **158**, 20–26 (2019).

52. J. Penfold, R. M. Richardson, A. Zarbakhsh, J. R. P. Webster, D. G. Bucknall, A. R. Rennie, R. A. L. Jones, T. Cosgrove, R. K. Thomas, J. S. Higgins, P. D. I. Fletcher, E. Dickinson, S. J. Roser, I. A. McLure, A. R. Hillman, R. W. Richards, E. J. Staples, A. N. Burgess, E. A. Simister, J. W. White, Recent advances in the study of chemical surfaces and interfaces by specular neutron reflection. *J. Chem. Soc. Faraday Trans.* **93**, 3899–3917 (1997).

53. J. R. P. Webster, S. Langridge, R. M. Dalgliesh, T. R. Charlton, Reflectometry techniques on the Second Target Station at ISIS: Methods and science. *Eur. Phys. J. Plus* **126**, 112 (2011).

54. L. A. Clifton, F. Ciesielski, M. W. A. Skoda, N. Paracini, S. A. Holt, J. H. Lakey, The effect of lipopolysaccharide core oligosaccharide size on the electrostatic binding of antimicrobial proteins to models of the gram negative bacterial outer membrane. *Langmuir* **32**, 3485–3494 (2016).

55. M. Born, E. Wolf, A. B. Bhatia, *Principles of Optics: Electromagnetic Theory of Propagation, Interference, and Diffraction of Light* (Cambridge University Press, ed. 7, 2019).

56. H. Haario, M. Laine, A. Mira, E. Saksman, DRAM: Efficient adaptive MCMC. *Stat. Comput.* **16**, 339–354 (2006).

57. D. S. Sivia, J. R. P. Webster, The Bayesian approach to reflectivity data. *Physica B* **248**, 327–337 (1998).

58. D. S. Sivia, J. Skilling, in *Data Analysis: A Bayesian Tutorial* (Oxford Univ. Press, ed. 2, 2006), pp. xii, 246.

59. B. Stuart, D. J. Ando, in *Biological Applications of Infrared Spectroscopy* (Analytical Chemistry by Open Learning, Published on behalf of ACOL (University of Greenwich) by John Wiley, Chichester, 1997), pp. xx, 191.
